# Large-scale gene network analysis reveals the significance of extracellular matrix pathway and homeobox genes in acute myeloid leukemia: an introduction to the Pigengene package and its applications

**DOI:** 10.1186/s12920-017-0253-6

**Published:** 2017-03-16

**Authors:** Amir Foroushani, Rupesh Agrahari, Roderick Docking, Linda Chang, Gerben Duns, Monika Hudoba, Aly Karsan, Habil Zare

**Affiliations:** 1 Department of Computer Science, Texas State University, 601 University Drive, San Marcos, USA; 2 Department of Pathology and Laboratory Medicine, British Columbia Cancer Agency, 675 West 10th Ave, Vancouver, Canada; 3 Department of Pathology and Laboratory Medicine, Vancouver General Hospital, 899 W 12th Ave, Vancouver, Canada

**Keywords:** Gene expression, Network analysis, Leukemia, Extracellular matrix, Homeobox, Hematological malignancy

## Abstract

**Background:**

The distinct types of hematological malignancies have different biological mechanisms and prognoses. For instance, myelodysplastic syndrome (MDS) is generally indolent and low risk; however, it may transform into acute myeloid leukemia (AML), which is much more aggressive.

**Methods:**

We develop a novel network analysis approach that uses expression of eigengenes to delineate the biological differences between these two diseases.

**Results:**

We find that specific genes in the extracellular matrix pathway are underexpressed in AML. We validate this finding in three ways: (a) We train our model on a microarray dataset of 364 cases and test it on an RNA Seq dataset of 74 cases. Our model showed 95% sensitivity and 86% specificity in the training dataset and showed 98% sensitivity and 91% specificity in the test dataset. This confirms that the identified biological signatures are independent from the expression profiling technology and independent from the training dataset.

(b) Immunocytochemistry confirms that *MMP9*, an exemplar protein in the extracellular matrix, is underexpressed in AML. (c) *MMP9* is hypermethylated in the majority of AML cases (*n*=194, Welch’s t-test *p*-value <10^−138^), which complies with its low expression in AML.

Our novel network analysis approach is generalizable and useful in studying other complex diseases (e.g., breast cancer prognosis). We implement our methodology in the Pigengene software package, which is publicly available through Bioconductor.

**Conclusions:**

Eigengenes define informative biological signatures that are robust with respect to expression profiling technology. These signatures provide valuable information about the underlying biology of diseases, and they are useful in predicting diagnosis and prognosis.

**Electronic supplementary material:**

The online version of this article (doi:10.1186/s12920-017-0253-6) contains supplementary material, which is available to authorized users.

## Background

Acute myeloid leukemia (AML) is an aggressive type of blood cancer and accounts for 1.2% of cancer deaths in the United States [1]. It is the most common acute leukemia, which is characterized by the rapid growth of immature white blood cells. These cells interfere with the production of normal blood cells in the bone marrow. Without treatment, AML can lead to death within months after diagnosis [2]. Myelodysplastic syndrome (MDS) are a set of less aggressive diseases; however, about 30 to 40% of MDS cases can transform into AML [3]. Therefore, it is critical to delineate the exact mechanisms of this transformation [4].

Possible molecular mechanisms include genetic mutations [5, 6], chromosomal abnormalities [7], and epigenetic changes [8, 9]. For example, mutation and abnormal expression of mRNA splicing genes such as *SRSF2* [10] and *SF3B1* [11] are associated with the prognosis of MDS. Overexpression of *Bcl-2* increases resistance of MDS cells to apoptosis [12], and it can play a role in the transformation into leukemia [13]. Similarly, the abnormal expression of some miRNAs such as miR-125 and miR-155 can lead to aberrant self-renewal of HSC [14], a characteristic of AML.

Although investigating the differences between AML and MDS at the molecular level has provided valuable insight, the research in this area has only scratched the surface of the problem. In particular, the current knowledge is far from adequate for the development of strategies for preventing or predicting the transformation of MDS into AML [9]. Researchers have proposed gene expression profiling as a systematic approach to explore the biology and clinical heterogeneity of MDS.

Most notably, Microarray Innovations in Leukemia (MILE), an international research consortium, assessed the clinical utility of gene expression profiling for the diagnosis and classification of leukemia subtypes [15, 16]. They investigated 3334 leukemia patients, including 202 AML with normal karyotype (AML-NK) and 164 MDS cases in their study, and they developed a classifier to distinguish MDS from AML. While their classifier could correctly predict 93% of AML cases from expression profiles, it failed to identify half of MDS cases [16]. This emphasized the heterogeneity of MDS and underlined the need for more sophisticated approaches for analyzing expression profiles. Specifically, the following challenges limited the performance of the classification:

- The classifier was based only on the 100 most differentially expressed genes. However, the biological processes in a hematopoietic cell often depend on the coordination of many more genes. Because the status of the cell is determined by the level of expression of hundreds of transcripts, restricting the analysis to only 100 genes could decrease the statistical power to a great extent [17]. Also, a random gene might be considered differentially expressed due to biological or technical noise or due to the difference in the analyzed cell types. Such a gene would convolute a classification based on differentially expressed genes [18].
- The produced data were inconsistent because of multiple platforms and approaches used across different institutions [9]. For instance, if a signature was defined using the level of expression in a microarray dataset, it would be very challenging to interpret and use that signature in an RNA-Seq dataset produced in a different laboratory [19].

We hypothesized that gene network analysis addresses both of the above challenges because it models the interactions between genes in a comprehensive structure [20, 21] (Additional file 1: Note S1). Recently, Liu reviewed the computational methods that employ a gene network approach to identify biomarkers from high-throughput data [22]. Gene networks provide a systematic way to organize complex data, and to identify biomarkers that are useful in improving diagnosis, prognosis and therapy of diseases.

**Fig. 1:**
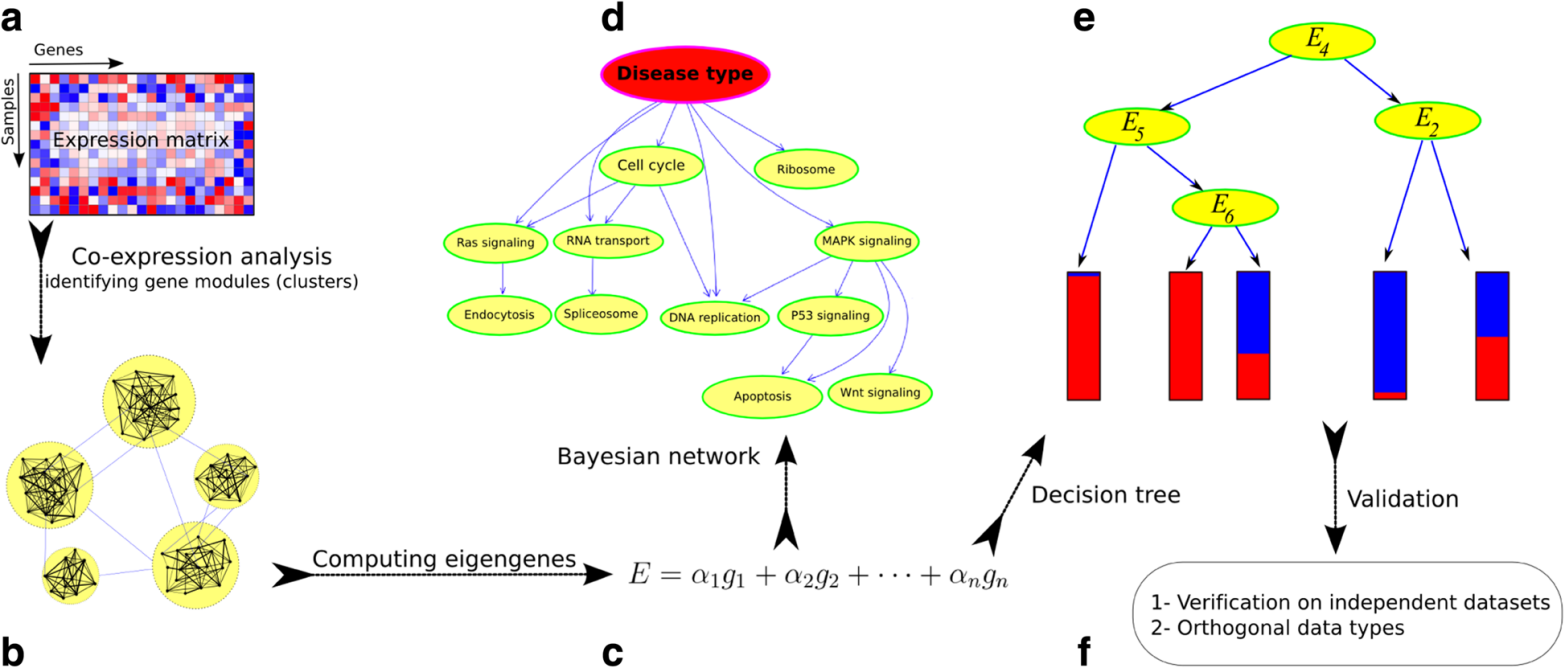
Schematic view of the Pigengene methodology. **a** The input is a gene expression profile (matrix) provided by RNA-Seq or microarray. **b** The coexpression network is built according to the correlation between gene pairs. **c** For each module, an eigengene is computed as a weighted average of the expression of all genes in that module. **d** Optionally, a Bayesian network is fitted to the eigengenes to delineate the relationships between modules. **e** A decision tree is fitted to the eigengenes and used for classification. **f** The results are validated on independent expression datasets and also evaluated using other data types. For instance, DNA methylation profiles can confirm gene-silencing events [43]

To address the above-mentioned challenges in analysis of expression profiles, we developed Pigengene, a novel methodology that is inspired by—and builds upon—coexpression network analysis and Bayesian networks. Briefly, we identify gene modules using coexpression network analysis [23]. We summarize the biological information of each module in one *eigengene* using principal component analysis (PCA) [24]. Our approach is fundamentally different from applying PCA directly on the entire expression profile, which can lead to significant loss of information [25]. We innovatively use eigengenes as biological signatures (features) to identify the mechanisms underlying the disease. For instance, we use eigengenes to train a Bayesian network that models the probabilistic dependencies between all modules. Alternatively, we infer a decision tree to predict the disease type based on eigengenes. The main idea of our methodology is illustrated in Fig. 1.

We used our methodology to classify patients in the MILE dataset. The accuracy of our model reached 95% for AML and 86% for MDS thus significantly outperforming the previously reported accuracy of 93 and 50%, respectively [16] (Table 1). To show the generalizability of the proposed approach, we report the results of applying it to several cohorts of breast cancer.

**Table 1:** The confusion matrices show the accuracy of our decision tree on the training (MILE) and test (BCCA) datasets

Dataset	MILE (train)		BCCA (test)	
Disease	AML-NK	MDS	AML-NK	MDS
Full tree (155 genes)	191 (95%)	141 (86%)	51 (98%)	20 (91%)
Reduced tree (14 genes)	181 (90%)	137 (84%)	51 (98%)	20 (91%)
Mills et al. [16]	188 (93%)	82 (50%)		
Reference diagnosis	202	164	52	22

The percentages of correctly identified cases with respect to the reference diagnosis are shown in parentheses. Compared to Mills et al., our decision tree is 36% more sensitive to MDS. The sensitivity to AML is comparable in both approaches

## Results

We identified 33 gene modules as clusters of genes that are coexpressed in the 202 AML cases from the MILE dataset [23] (Additional file 1: Note S2). The sizes of the modules vary in the range of 21 to 888, with a mean and median of 153 and 75, respectively (Additional file 1: Figure S1).

### Analysis of gene modules

Overrepresentation analysis reveals that some of the modules are associated with canonical pathways and biological processes. For instance, module 6 is enriched with genes that are related to the cell cycle. That is, out of 421 genes in the Reactome cell cycle pathway [26], 81 (19%) are grouped in module 6, which consists of 255 genes (*p*-value of the hypergeometric test <10^−37^). Similarly, module 12 is associated with extracellular matrix, module 14 with cytotoxic pathway (CD8+ T cells), module 15 with DNA replication, and module 21 with translation (Additional file 1: Figures S2 and S3 and Additional file 2: Table S2).

Module 33 is the smallest module containing 21 genes. We named it HIST1 because almost all of its genes (20, 95%) encode proteins from the linker histone, or H1, family (Additional file 3: Table S1). Half of the 39 genes in module 28 are from the homeobox family. Considering that this module contains 10 *HOXA* and 9 *HOXB* genes, we named it HOXA&B module. It is highly enriched with the homeobox genes that have been reported to be associated with the development and prognosis of AML [27, 28] (Additional file 1: Figure S4, Additional file 3: Table S1 and Additional file 4: Table S3).

### Eigengenes are associated with the disease

We summarized the biological information of each module in one eigengene (Additional file 5: Table S4). An eigengene of a module is a weighted average of expression of all genes in that module. The weights were adjusted such that the loss in the biological information is minimized (Methods) [24, 29]. In the MILE dataset, all module eigengenes present significantly different expression in AML vs. MDS. The adjusted Welch’s t-test *p*-values are in the range of 10^−61^ to 10^−6^, with a median of 10^−24^ (Additional file 1: Figure S5) [30].

**Fig. 2:**
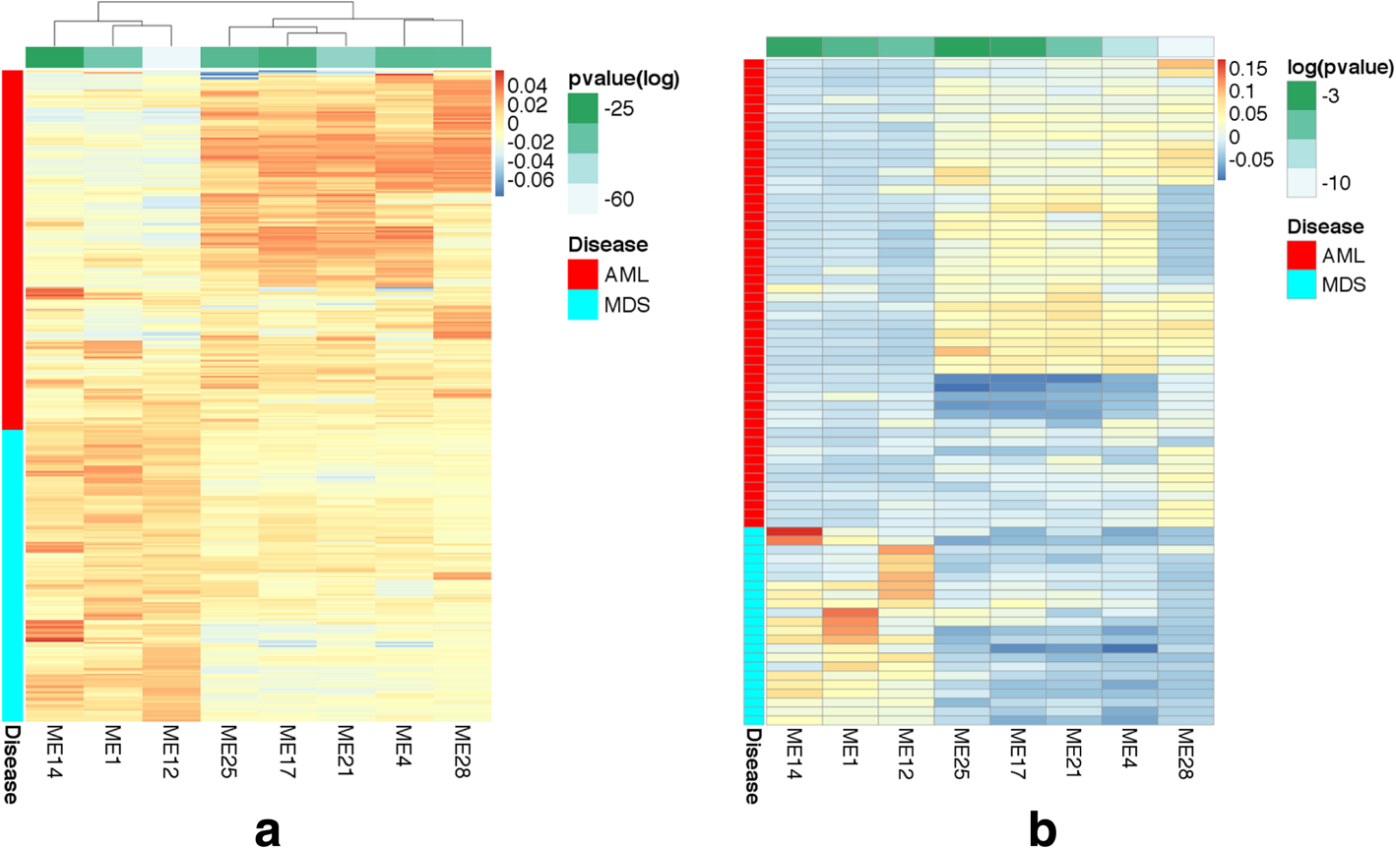
The eight eigengenes that are differentially expressed in both datasets. We computed an eigengene for each module as a weighted average of the expression of all genes in that module. The intensity of the colors in each heatmap corresponds to the normalized average expression. **a** Each column corresponds to an eigengene. Each row shows the expression of a case from the MILE microarray dataset. AML cases were first clustered and then plotted together with MDS cases for comparison. Three (five) eigengenes have clearly higher expression in MDS (AML) cases. Table 2 reports the adjusted *p*-values, shown in the *green strips at the top*. **b** The corresponding expression values are similar in the BCCA RNA-Seq dataset, which indicates that these biological signatures are robust and independent from the profiling platform

We hypothesized that the eigengenes are important biological signatures that can predict the disease type solely based on gene expression. To validate this hypothesis, we developed an innovative approach to infer the values of these eigengenes using the RNA-Seq data from an independent dataset produced at the British Columbia Cancer Agency (BCCA) (Methods). We used this approach to investigate the patterns common in the MILE and BCCA datasets. Interestingly, eight eigengenes achieve significant *p*-values (<0.01, Bonferroni adjusted) on both BCCA and MILE datasets, indicating that these biological signatures are independent from the profiling platform (Fig. 2 and Table 2). The eight differentially expressed modules include module 28 (HOXA&B), module 21 (translation), module 12 (extracellular matrix), and module 14 (CD8+ T cells).

**Table 2:** These eigengenes were differentially expressed in AML vs. MDS cases

Module	1	4	12	14	17	21	25	28
*P*-value (MILE)	10^−37^	10^−32^	10^−61^	10^−23^	10^−28^	10^−43^	10^−32^	10^−33^
*P*-value (BCCA)	10^−3^	10^−5^	10^−3^	10^−2^	10^−3^	10^−7^	10^−3^	10^−11^

They had adjusted *p*-values (Welch’s t-test) less than 0.01 in both the MILE and BCCA datasets

**Fig. 3:**
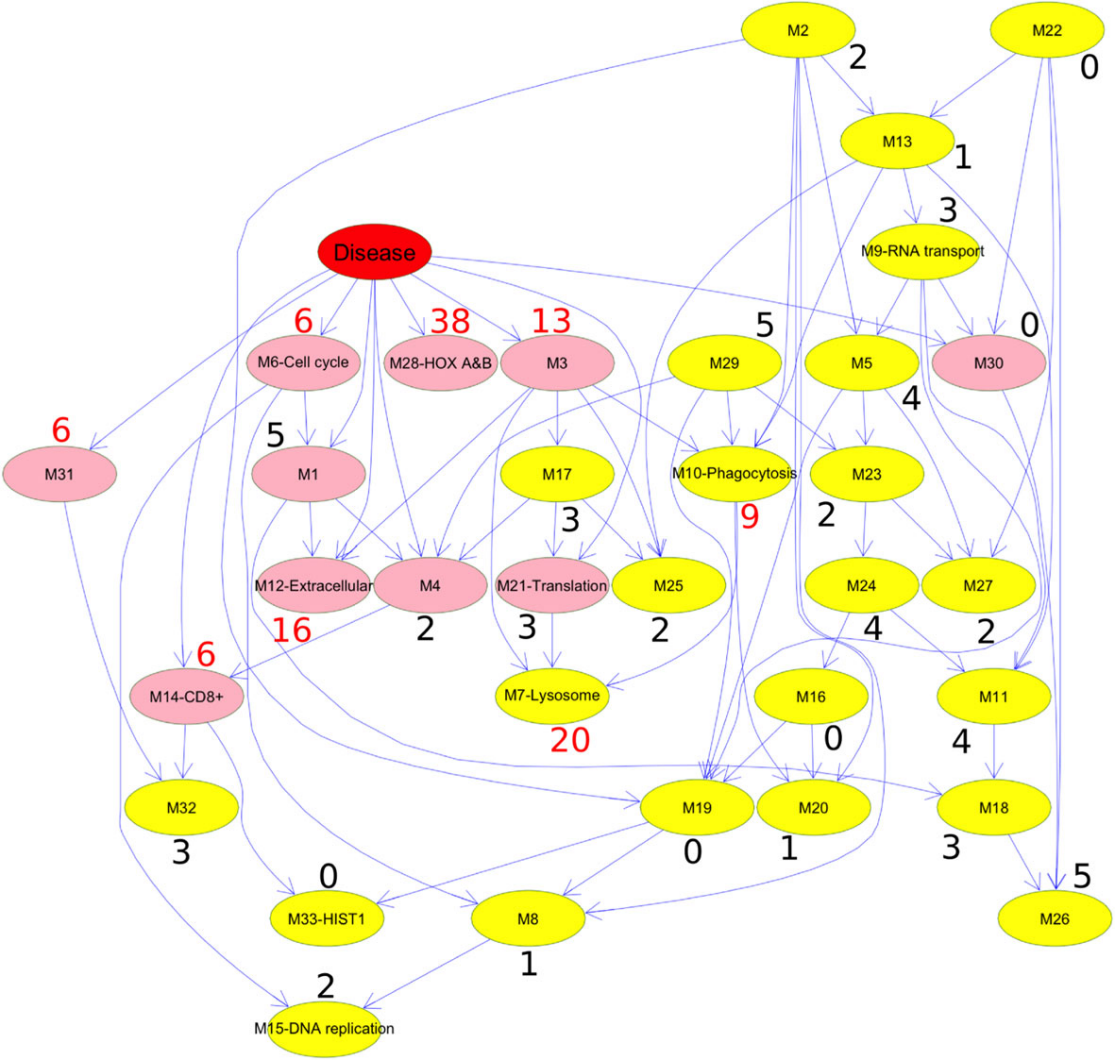
The Bayesian network fitted to the eigengenes. Each node represents an eigengene of a module. The arcs model the probabilistic dependencies between the modules [86]. The “Disease” node is set to 1 for AML and 0 for MDS, and its children are highlighted in *pink*. Some modules are labeled based on their association with a biological process or a pathway (Additional file 2: Table S2). We used Miller et al., survey to identify the 427 genes reported to be associated with AML in at least three studies [81] (Additional file 3: Table S1). For each module, the percentage of AML-related genes is noted. The percentages that exceed 5% are shown in *red*. As expected, most of the children of Disease are enriched in genes known to be associated with AML. Specifically, the average of percentages over the children of the Disease node is 10%, which is twice the average of all modules (5%). Also, hypergeometric tests showed that modules 3, 7, 12, and 28 are statistically enriched with AML-related genes (Bonferroni adjusted *p*-values are 10^−7^, 10^−13^, 10^−3^, and 10^−8^, respectively). All four of these modules are descendants of the Disease node

We fitted a Bayesian network to the eigengenes to determine the relationships of the modules with each other and with the type of hematological malignancy (Additional file 1: Note S3) [31]. Descendants of the “Disease” node, the variable that models the type of malignancy, are enriched with genes known to be associated with AML (Fig. 3). The relatively high dependency between these eigengenes and the disease type suggests that they have useful biological information that can explain the differences between the two diseases.

### AML and MDS are different in their expression of extracellular matrix, *HOXA*, and *HOXB* genes

**Fig. 4:**
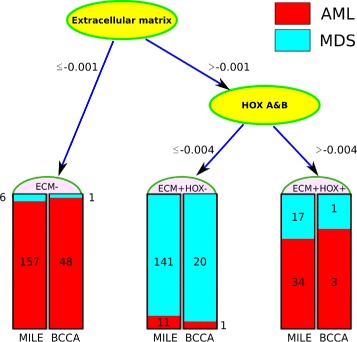
A simple decision tree for distinguishing AML from MDS cases. If the normalized extracellular matrix eigengene of a case is less than -0.001, it is classified as AML. Otherwise, the HOXA&B eigengene determines whether the case is AML (>−0.004) or MDS (≤−0.004). The number of cases classified in each leaf is noted for both the MILE (*left*) and the BCCA (*right*) datasets. Only the middle leaf corresponds to MDS. At the fixed thresholds shown above, this tree correctly classified 328 cases (90%) in the MILE dataset (the training set) and 71 cases (96%) in the BCCA dataset (the test set)

We fitted a decision tree to the eight children of the Disease node in our Bayesian network (R package C50 version 0.1.0-24) [32]. We used only MILE data to infer the topology of the tree and the corresponding parameters. The algorithm *automatically* selected the extracellular matrix and HOXA&B eigengenes (modules 12 and 28, respectively). The inferred decision tree had high predictive accuracy (Fig. 4). Specifically, 191 AML-NK cases (95%) and 141 MDS cases (86%) were correctly identified (Additional file 6: Table S5).

**Fig. 5:**
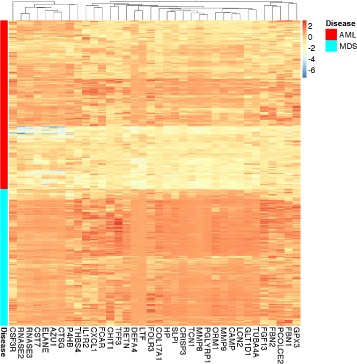
Comparing the expression of extracellular region genes. Each column shows the expression of a gene from the extracellular matrix module that is associated with the “extracellular region” in the cellular component category of Gene Ontology (GO). For clarity, each column is scaled by subtracting its mean and dividing by its standard deviation. Each row corresponds to a sample from the MILE dataset. These 36 genes are generally underexpressed in AML compared to MDS. The expression of all 133 genes in the extracellular matrix module have a similar pattern (Additional file 1: Figure S6)

**Fig. 6:**
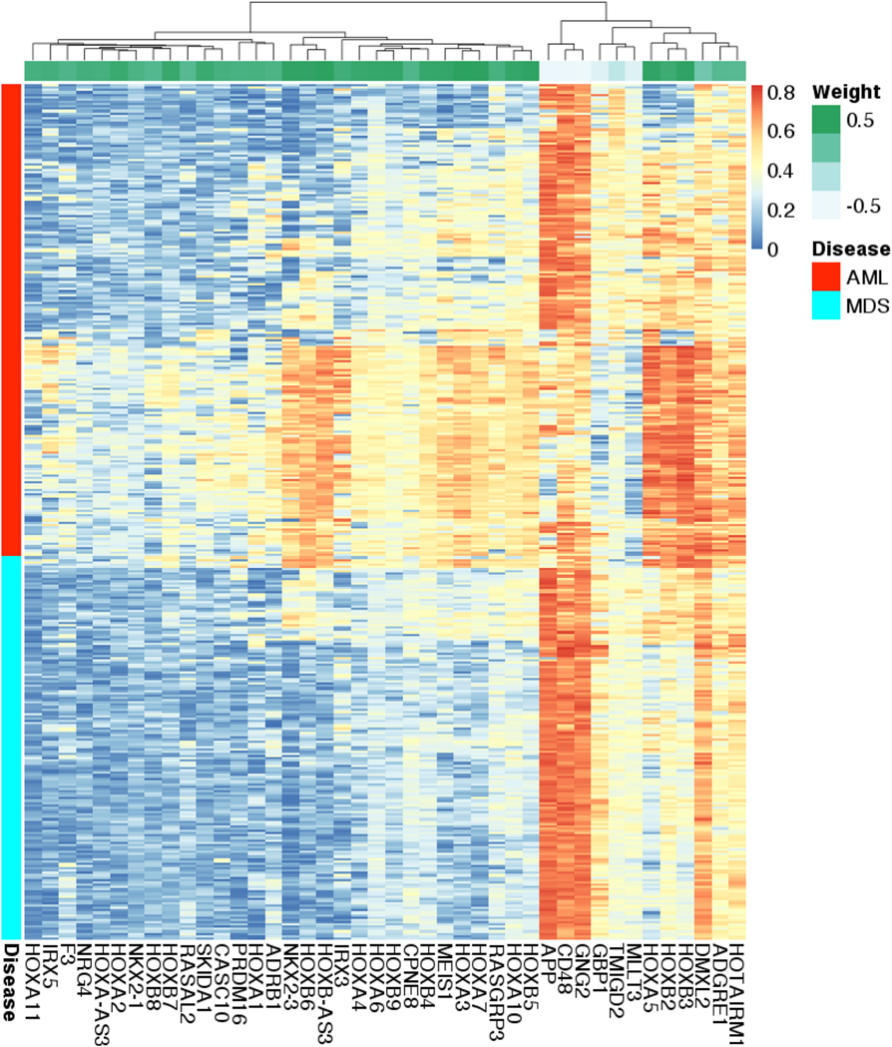
Comparing the expression of genes in the HOXA&B module. Expression of every member of HOXA&B module is shown in one column. Each row corresponds to a sample from the MILE dataset. The majority of *HOXA* and *HOXB* genes in this module are not expressed in MDS. Their expression in AML are variable indicating the heterogeneity of the disease. They anticorrelate with *GNG2*, *CD48*, and *APP*, which have the least negative weight (-0.7) in the corresponding eigengene (the *green strip at the top*). These patterns are similar in the BCCA dataset (Additional file 1: Figure S7)

The majority of AML cases (157, 78%) were identified because of their low expression of extracellular matrix genes (i.e., their normalized eigengene value was less than −0.001). For the rest of the cases, which expressed the extracellular matrix eigengene, the tree considered the expression of the HOXA&B eigengene. If it was over −0.004, the case was classified as AML. The tree shows that for a case to be MDS, it must have relatively high expression of the extracellular matrix (Fig. 5 and Additional file 1: Figure S6) and low expression of HOXA&B (Fig. 6 and Additional file 1: Figure S7).

#### Misclassification of MDS was associated with risk factor

The International Prognostic Scoring System (IPSS) score [3] is the standard tool for MDS risk stratification [33]. It ranges from 0 to 3.5, and a higher value indicates a poorer prognosis. There are 30 MDS cases (18%) in the MILE dataset with poor prognosis (IPSS ≥1.5). This set has a significant overlap with the 23 cases “misclassified” by our decision tree (Additional file 6: Table S5). Specifically, 15 MDS cases with poor prognosis show AML signatures and are classified as AML by the tree (hypergeometric test *p*-value <10^−7^). This suggests that underexpression of the extracellular genes and overexpression of the *HOXA* genes in an MDS case can be considered as a risk factor. Because transition into AML is more likely for such an MDS case, a monitoring assay can be developed based on these signatures.

#### Validating AML signatures in an independent dataset

We validated the performance of the tree on classifying 74 cases in the BCCA dataset. To this end, we inferred the values of extracellular matrix and HOXA&B eigengenes in the BCCA dataset (Methods). With the same above-mentioned thresholds that performed well for the MILE dataset, the tree correctly identified 51 (98%) of the AML-NK and 20 (91%) of the MDS cases. The high accuracy of our decision tree was helpful in correcting a clerical error in annotating the dataset. In particular, two BCCA cases (B118 and B129), originally labeled with MDS, have signatures very similar to AML (Additional file 5: Table S4). Interestingly, a second review revealed that their correct diagnosis is in fact tAML (therapy–related AML) and AML–M1, respectively.

Although the decision tree was trained using only AML-NK subtype in the MILE dataset, its performance in differentiating some other subtypes of AML from MDS in the BCCA dataset is remarkable. In particular, all of the four AML-t(8;21) cases (100%), all of the four AML cases with complex karyotype cases (100%), all of the four AML cases with 11q23 abnormality (100%), and 9 out of 11 AML-inv(16) cases (82%) are all correctly classified as AML. However, cases from other subtypes, such as AML-t(15;17), AML-M6, and tAML, do not always show strong extracellular or HOXA&B signatures of AML-NK and are frequently misclassified as MDS (Additional file 6: Table S5). This is expected, because these three subtypes of AML are distinct and too different from AML-NK. In particular, leukemic cells in AML-t(15;17) and AML-M6 are relatively more differentiated [34], and may produce some extracellular matrix proteins.

### A minimal gene set for clinical testing

Considering the good performance of the decision tree, it is useful to develop a clinical test based on gene expression. The extracellular matrix and HOXA&B modules contain 113 and 42 genes, respectively. To infer the corresponding eigengenes, the expression of 155 genes are needed in total. If the number of genes is reduced without significant loss of accuracy, the test will be easier to use in clinical settings. Because the genes are correlated with each other in each module, shrinking the tree is expected to have little—or no—effect on the accuracy of classification.

Using a greedy approach, we excluded the majority of the 155 genes, and obtained a decision tree that need the expression values of only 14 genes (9%) (). The performance of the reduced tree is comparable to the original tree (Table 1). On the training set, the accuracy dropps by only 5% for AML and by 2% for MDS. On the test set, however, the reduced tree is as accurate as the full tree (Additional file 6: Table S5).

**Fig. 7:**
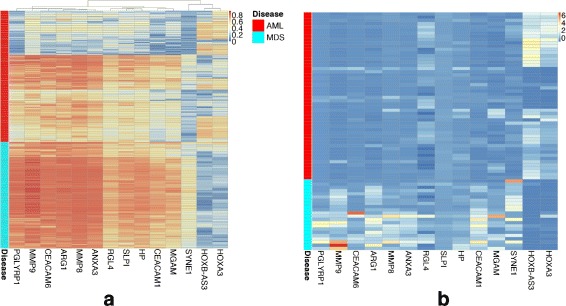
Comparing the expression of the 14 genes used in the reduced tree. Expression of the 14 genes that contribute to the reduced (compact) decision tree in the **a** MILE and **b** BCCA datasets are shown. The 12 extracellular matrix and the two *HOXA* genes are generally silenced and overexpressed in AML, respectively

The list of 14 genes used in the reduces tree included *PGLYRP1*, *MMP9*, *CEACAM6*, *ARG1*, *MMP8*, *ANXA3*, *RGL4*, *SLPI*, *HP*, *CEACAM1*, *MGAM*, *SYNE1* from the extracellular matrix module, and *HOXB-AS3* and *HOXA3* from HOXA&B module (Fig. 7).

### The significance of the extracellular matrix pathway in AML

The relationship between *HOX* genes and AML and their role in leukemogenesis are extensively studied [27, 27, 28]. Researchers have also explained the significance of the extracellular matrix pathway in the prognosis of cancers in general [35]. However, its role in the development of AML and other leukemias is more complicated. In addition to regulating cell growth [36], proliferation [37], differentiation [38], and apoptosis [39], it also mediates the migration of hematopoietic stem cells through the vessels [40]. Module 12 is enriched with extracellular matrix genes (Additional file 1: Figure S8). We investigated these genes, which defined a significant signature in our decision tree (Fig. 4).

Gene Ontology Cellular Component (GO-CC) analysis showed that 36 of 113 genes in module 12 code for proteins in the extracellular region (Additional file 1: Figure S8 and Additional file 7: Table S7). Moreover, 77 of the genes in this module are associated with at least one of the following categories: extracellular vesicular exosome (44 genes), extracellular region (36), extracellular space (30 genes), and plasma membrane (31 genes). We noted that 18 genes (16%) are located on chromosome 19. Almost all of these 113 genes are underexpressed in AML (Fig. 5 and Additional file 1: Figure S6). The enriched biological processes include: immune system process (adjusted *p*-value <10^−9^), killing by host of symbiont cells (<10^−3^), killing of cells in other organism involved in symbiotic interaction (<10^−2^), defense response to fungus (<10^−3^), antibacterial humoral response (<10^−2^), extracellular matrix disassembly (<10^−2^), and response to lipopolysaccharide (<10^−2^) (Additional file 8: Table S9) [41].

One particularly interesting gene from this module was *MMP9*, which had a relatively high contribution to the eigengene. Its weight is 0.92, the highest in the extracellular matrix pathway (Reactome [42]), and the eighth in the module (Additional file 7: Table S7). *MMP9* is a member of the matrix metalloproteinase (*MMP*) family, which has 23 members.

They remodel and degrade the extracellular matrix by cleaving its components [42]. In addition to *MMP9*, this module includes two other members of *MMP* family, namely *MMP8* (weight = 0.91) and *MMP25* (weight = 0.87). All of these three genes are underexpressed in AML (Additional file 1: Figure S9a). One way to confirm that these genes are silenced in AML would be to check epigenetic factors such as DNA methylation, which generally anticorrelates with gene expression [43]. We compared 194 AML cases of Acute Myeloid Leukemia (LAML) dataset from The Cancer Genome Atlas (TCGA) with 368 control cases, and we confirmed that these three genes were heavily methylated in AML (Additional file 1: Figure S9b and Additional file 9).

### Validating gene expression changes at the protein level

**Fig. 8:**
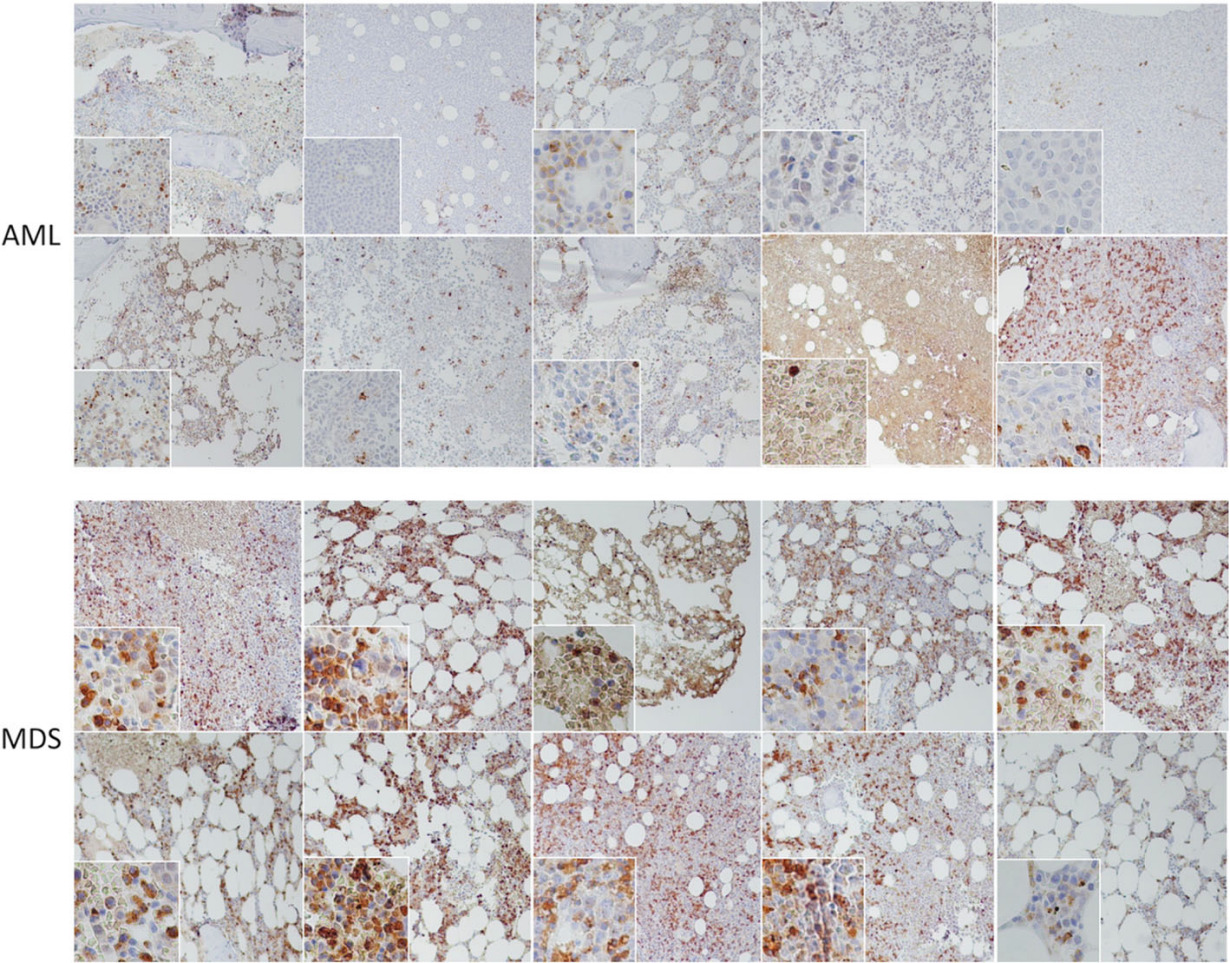
Expression of *MMP9* in AML and MDS. The bone marrow of 10 patients with AML (*upper panels*) and 10 patients with MDS *lower panels*) was immunostained in parallel with an *MMP9* antibody. *MMP9* expression is reduced to absent in AML blasts. Where staining is seen in AML, it is only present in mature myeloid cells but not leukemic cells

Given the strong discriminating capability of extracellular matrix gene expression in differentiating AML from MDS (Fig. 4), we attempted to determine whether a simple immunohistochemical stain would provide such a differentiation. We selected *MMP9* to test this, as it provided the highest-weighted contribution to the eigengene (0.92) within the extracellular matrix set of genes. We obtained 10 previously diagnosed AML cases and 10 previously diagnosed MDS cases, and performed immunostaining on the diagnostic bone marrow biopsies. As seen in Fig. 8, *MMP9* staining is drastically lower in the AML samples compared to the MDS cases.

### Validating the identified coexpression pattern in other AML-related datasets

The 113 genes in the extracellular matrix module are correlated and underexpressed in AML. To validate that the observed coexpression pattern is specifically associated with AML, we investigated the expression of these 113 genes in a large collection of human datasets. Specifically, we used Search-Based Exploration of Expression Compendium (SEEK) [44] to objectively compare the coexpression of these genes across a collection of 5210 datasets. SEEK automatically scored and ranked the datasets based on the significance of coexpression of our 113 genes. SEEK also computed empirical *p*-values to assess the statistical significance of scores. Specifically, random scores for each dataset was computed based on 5000 queries of 113 random genes, and a *p*-value was reported as the fraction of random scores that were higher than the reported score. The collection contains 61 AML-related datasets (1.2%), which mostly score high in the ranked list (Additional file 10: Table S8). In particular, all of the five top datasets are related to AML (GEO accession numbers: GSE15434 [45], GSE16015 [46], GSE12417 [47], GSE21261 [48], and GSE30599 [49]; with 251, 107, 405, 96, and 29 samples, respectively). The coexpression scores are 0.31, 0.29, 0.28, 0.28, and 0.27, respectively; and the adjusted empirical *p*-values are smaller than 10^−37^ for each of these five datasets. A hypergeometric test confirmed that the coexpression of the queried genes is significantly associated with AML (*p*-value <10^−9^). Thereby, our unbiased and objective SEEK analysis indicates that these genes define an expression signature that is specific to AML.

### Generalizability to studying other cancers

The described pipeline can also be applied to analyze other types of cancers and answer different biological questions. To demonstrate this, we applied our approach to a prognostic question in breast cancer research. Is it possible to identify low-risk breast cancer cases based solely on gene expression and thereby avoid overtreating a subset of patients who likely would not benefit from the additional toxic therapy [50]? In this type of prognostic setting, the emphasis lies on achieving very high specificity for predicted *low-risk* cases. For instance, the TRANSBIG Consortium [51] considers a test to be clinically practicable and reliable for ER+ breast cancer only if at least 88% of cases classified as low-risk have more than a 10-year overall survival. However, the only clinical test with such high precision is Oncotype DX, which is applicable to only one clinical subtype of breast cancer, stage I ER+ tumors [50]. Unfortunately, this method cannot be generalized to other breast cancer subtypes [52].

We analyzed 1374 ER+ cases from three datasets to train and validate our model (Methods and Additional file 1: Note S4). The low-risk specificity of our model is above 89% for all three datasets (Table 3 and Additional file 1: Figure S11). Two modules with 319 and 193 genes, respectively, were automatically selected (Additional file 11: Table S6). The larger module is associated with the mitotic cell cycle (*p*-value <10^−71^) and chromosome segregation (*p*-value <10^−28^). This module has 16 genes in common with the genes in the PAM50 assay, which is widely used in clinical settings to identify breast cancer subtypes [53]. These common genes include *UBE2T, BIRC5, CCNB1, CEP55, MELK, UBE2C, CENPF, PTTG1, EXO1, ANLN, CCNE1 CDC20, MKI67, KIF2C, MAPT,* and *FGFR4*. This is a significant overlap (*p*-value of the hypergeometric test <10^−10^).

**Table 3:** Accuracy of predicting breast cancer risk

Dataset	METABRIC discovery		METABRIC validation		MILLER (test)	
	Low risk	High risk	Low risk	High risk	Low risk	High risk
Predicted low risk	157 (**94%**)	11 (7%)	107 (**89%**)	13 (11%)	68 (**93%**)	5 (7%)
Predicted medium risk	278 (68%)	134 (33%)	236 (70%)	99 (30%)	55 (68%)	26 (32%)
Predicted high risk	21 (35%)	39 (65%)	33 (42%)	46 (58%)	29 (62%)	18 (38%)

The confusion matrices show the performance of our decision tree on three datasets. The percentage of predicted cases with respect to the total number of predictions in each group is shown in parentheses. From a clinical standpoint, it is important to achieve a high precision (positive predictive value) for low risk cases (shown in bold) to confidently recommend a less agressive treatment regimen for a subset of patients. The probability of surviving more than 10 years is above 89% for the predicted low risk cases in all the three datasets (Additional file 1: Figure S11)

The smaller module is associated with *translational control* (Additional file 1: Figure S12). The expression of the majority of the genes (122, 63%) is correlated with poor prognosis. Notable genes include *AKT1, GSK3B, MTOR, RAF1*, and *SRC* from the epidermal growth factor receptor (ErbB) signaling pathway [54]. In contrast, the high expression of 71 genes (37%) in this module—including 16 ribosome-related genes such as *RPL22, RPL26, RPS27, RPS27A, RPL13A, RPL21* and *RPLP0*—correlate with good prognosis. This may be predicted, as the loss of function or abnormal expression of proteins involved in ribosomal biogenesis is associated with activation of the tumor suppressor p53 pathway [55, 56]. A possible mechanism of p53 activation could be through binding free (non-ribosome-bound) ribosomal proteins with *MDM2*, which modulates the inhibitory activity of *MDM2* on *p53* [55].

None of the 193 genes from the smaller module is in common with PAM50. This suggests that the corresponding eigengene can be considered as a novel biological signature to assess breast cancer prognosis, and it can be a basis for improving clinical tests. Overall, our model is biologically plausible because regulated cell cycle and controlled translation are generally associated with better prognostic outcome [57].

## Discussion

Biological processes in a cell often require coordination between *multiple* genes and proteins, not just one gene or a single protein. Accordingly, we used network analysis to delineate the differences in gene expression profiles of AML and MDS in a systematic and robust way (Additional file 1: Note S1). We compared the expression at the module level to minimize the effect of artifacts such as a random change in expression of an isolated gene and other biological or technical noise (Additional file 1: Figure S10).

The results of our study underline the association of the extracellular matrix pathway with AML, and also confirm that the overexpression of homeobox genes is a biological characteristic of AML. These two signatures are biologically related [58]. Homeobox genes encode transcription factors that regulate the development of body structures during the embryonic period [59]. They also have key roles in adult tissue remodeling and pathogenesis [60]. In particular, specific homeobox genes can regulate the extracellular matrix through the expression of matrix-degrading proteinases [61]. For instance, the expression of the *HOXA3* and *HOXB3* are upregulated during wound healing to remodel the extracellular matrix and to increase endothelial cell migration [62]. Overexpression of *HOXA7*, which is associated with poor prognosis of AML [63], can modify the interactions between hematopoietic progenitor cells and the extracellular matrix in the bone marrow. This alteration can be responsible for blocking the differentiation process in AML cells [64].

The two signatures are highly associated with AML to such a degree that they can be used to design an accurate clinical test for differential diagnosis between AML and MDS. Furthermore, the following confirmatory evidence supports our findings on the significance of the extracellular matrix pathway in AML:

- Our decision tree can accurately predict the diagnosis in a validating dataset (BCCA) without the need to change the parameters that were fitted to the training dataset (MILE). Our results confirm that the model was not overfitted to the training dataset.
- SEEK analysis confirms that the genes in the extracellular matrix module are coexpressed in several other AML-related datasets.
- The three *MMP* genes in the extracellular matrix, *MMP9*, *MMP8*, and *MMP25*, are methylated in AML.
- Immunocytochemistry showed that *MMP9* is underexpressed in AML at the protein level.

*MMP9* is an important gene in our analysis, and it has a distinct expression profile between the two diseases. *MMP9* acts as a cell surface transducer by cleaving the extracellular matrix and other proteins, including chemokines, cytokines, and growth factor receptors. In this way, it can regulate key signaling pathways in cell growth, migration, invasion, inflammation, and angiogenesis [65]. While *MMP9* was previously reported to have a critical role in AML invasion and metastasis [66–69], the relationship between its expression and the prognosis of hematological malignancies is complicated. For instance, Aref et al. report that 43 pretreatment AML cases had significantly lower expression of *MMP9* as compared to 10 controls. However, after chemotherapy, *MMP9* was expressed significantly higher in relapsed cases as compared to complete remission cases [70].

In this context, the high expression of *MMP9* in MDS, which we showed is more than AML, is interesting. Correspondingly, Travaglino et al. measured *MMP2* and *MMP9* in myeloid cells of 143 MDS cases using immunocytochemistry. They found that high *MMP* levels are associated with longer overall survival [71]. One possible interpretation is that by deregulating the extracellular matrix, *MMP9* may interrupt the survival signalling in MDS and lead to apoptosis. In contrast, lowering *MMP9* expression may prolong the life of the MDS cells and facilitate the transition into AML. *MMP9* processing of the matrix may also have an impact on blast cell invasion, dissemination, and homing [70]. However, functional studies will be needed to determine the mechanism and impact of *MMP9* on myeloid cancers. A competing theory would be that the observed differences in the extracellular matrix activity might be due to differences in the underlying cell-types.

Our approach has novel methodological contributions to gene expression analysis. While other scholars have used weights (loadings) of eigengenes to study genes in a module [24], we are the first to use values of eigengenes directly as biological signatures. We developed an approach to infer and compare eigengenes across datasets. Our approach is fundamentally different from applying PCA directly on the entire expression profile, which is not a promising approach because the first few PCs may not have enough information on the modules’ structure [25].

An analysis based on a limited number of genes with the best *p*-value can be convoluted by random, dramatic expression changes due to biological or technical noise [17]. In contrast, because an eigengene is a weighted *average* expression of several genes, our systematic and holistic approach is much more robust than the alternative approaches that select one or a few genes from each module [72, 73]. We show that our methodology is generalizable and useful in studying other malignancies by applying it to several breast cancer datasets.

## Conclusions

Eigengenes are robust informative biological signatures. They are useful in predicting the diagnosis and prognosis, and also, in delineating the molecular characteristics of diseases. For instance, we used large-scale network analysis to show that underexpression of particular genes in the extracellular matrix pathway is a specific characteristic of AML.

## Methods

### The AML gene expression datasets

We downloaded the expression profiles of 202 AML-NK and 164 MDS cases from Gene Expression Omnibus (GEO) (series number GSE15061) [16], Additional file 12. The dataset is part of the expression MIcroarray analysis for diagnosis of LEukaemia (MILE) series. For simplicity, we refer to this expression profile as the *MILE dataset*, which was used to train our model. To validate our model and test the accuracy of classification, we used RNA-Seq data from 133 AML and 22 MDS cases analyzed at British Columbia Cancer Agency. For simplicity, we refer to this expression profile as the *BCCA dataset*, which is independent from the MILE dataset. From the 133 AML cases, 52 were AML-NK and thus were comparable with the 202 cases from the MILE dataset (Additional file 6: Table S5). We used Sailfish (version 0.6.3) [74] to compute reads per kilobase per million mapped reads (RPKM) values [75] for each gene, and considered the natural logarithm of RPKM to measure gene expression.

### Breast cancer datasets

We used 640 ER+ cases from the Molecular Taxonomy of Breast Cancer International Consortium (METABRIC) [76] discovery dataset for training. We evaluated the resulting model on 533 different cases from the METABRIC validation dataset. We also validated the prognostic value of the inferred biological signatures using 201 cases from a second independent dataset produced by Miller et al. [77] (GEO accession number GSE3494). The details of our analysis on these three datasets is presented in Additional file 1: Note S4.

### Detailed description of the Pigengene methodology

**Preprocessing** The input to the Pigengene methodology includes two gene expression profiles corresponding to two biological conditions (e.g., AML and MDS in this paper). Optionally, the user can provide a validating dataset (e.g., BCCA dataset). The train and validation datasets do not need to be assayed using the same platform. Thas is, one dataset can be microarray and the other one can be RNA-Seq. Figure 1 shows the main steps of the Pigengene methodology. More specificity, the first step of the analysis is to exclude the genes that have too little variation or negligible expression across the conditions. This can be done using a differential expressed analysis, which computes a *p*-value for each gene with the null hypothesis that it is similarly expressed in the two conditions. Consistent with the common approach in the gene network analysis [78, 79], we kept only the top one-third genes with the best *p*-values in our analysis.

**Constructing the coexpression network:** We used the WGCNA package to construct a coexpression gene network, in which each node (vertex) is a gene and the edge (connection) between two genes is weighted based on the correlation between their expression values (Additional file 1: Note S2). WGCNA uses a hierarchical clustering approach to identify gene modules from the coexpression network.

**Computing eigengenes:** We used principal component analysis (PCA) to compute an eigengene for each module. First, we balanced the number of AML and MDS cases using oversampling, so that both disease types had comparable representatives in the analysis. Specifically, we repeated the data of each AML and MDS case 9 and 11 times, and obtained 1818 and 1804 samples from each type, respectively. Then, we applied the moduleEigengenes() function from the WGCNA package on the oversampled data. We ran it with the default parameters, and computed an eigengene for each of the modules identified earlier. This function computed the first principal component of each module, which maximized the explained variance ensuring the loss in the biological information was minimized. [24, 29] (Additional file 5: Table S4).

**Inferring the decision tree:** We use eigengenes as features to infer a decision tree (R package C50 version 0.1.0-24) [32]. While the C50 package uses a heuristic approach to select the best set of features, its default arguments does not result in optimal performance when too many features are provided. The solutions include: 1) using a Bayesian network to determine the relationships of the modules with each other and with the type of hematological malignancy (Additional file 1: Note S3) [31], 2) using a feature scoring algorithm such as FeaLect [80], and 3) adjusting the C50 parameters, for example, enforcing the number of samples in each node to be at least 10%. The first and the third solutions are implemented in the Pigengene package through the bnNum argument of the one.step.pigengene() function and the minPerLeaf argument of the make.decision.tree() function, respectively. These two approaches resulted in the same decision tree presented in this paper (Fig. 4).

**Inferring the values of eigengenes in an independent dataset:** When a validation dataset is available (i.e., the BCCA dataset in our study), the values of the eigengenes need to be inferred in the validation dataset. We computed eigengenes using the MILE dataset, which is a microarray dataset. It was challenging to compute the values of the same eigengenes for BCCA cases because the BCCA dataset was produced using a different platform (i.e., RNA-Seq) [19]. The simple approach of applying PCA on the BCCA data would fail; It would result in different weights (loadings), and the eigengenes would not be comparable between the two datasets. Instead, we inferred the values of the eigengenes for BCCA cases using the same weights obtained from the MILE dataset. Specifically, for each module, we identified the genes that are common in both datasets. Then, we scaled the expression of those genes by subtracting their mean and dividing by their standard deviation. We used the scaled expression values to compute the eigengene (the weighted average of expression) for each BCCA case. The project.eigen() function from our Pigengene package facilitates this approach.

**Reducing the number of genes needed for the decision tree:** Our decision tree used the eigengenes of HOXA&B and extracellular matrix modules, which were weighted averages of the expression of 42 and 155 genes, respectively. To reduce the number of genes, we repeated the following greedy procedure [72]: We excluded the gene with the lowest absolute weight, inferred the eigengenes using the remaining genes, and used the updated eigengenes as input to the decision tree. In each iteration, we used the same tree structure and thresholds, and we measured the accuracy of classification. We repeated this procedure until the tree needed only 14 genes, because excluding any more genes would result in a significant decline in the accuracy of the classification. The sufficiency of these 14 related genes indicates that they contain the core biological information needed for classification. The compact.tree() function from our Pigengene package facilitates this approach.

### Code availability

“Pigengene”, a documented R package that implements our approach, is publicly available through Bioconductor: http://bioconductor.org/packages/Pigengene. The results presented in this paper can be reproduced using version 0.99.19. To apply our methodology in other studies, we strongly recommend using the most recent version. We encourage users to use the Bioconductor mailing list to send bug reports and seek technical help.

## Additional files

Additional file 1 Supplementary Notes and Figures. (PDF 1656 kb)

Additional file 2
**Table S2.** Overrepresented pathways. The canonical pathways that are overrepresented in the modules are available as part of the online supplementary materials. Each sheet in the excel file corresponds to a module. The statistics for each pathway (gene set) is reported on one row, in particular, the *p*-value of a hypergeometric test with the null hypothesis that the genes from the pathway were observed in the module by chance. The other columns include the name of the pathway (Set Name), the number of genes observed in the module (In Module), the number of the genes from the pathway that are present in our pool of 9,166 genes (Set Size), percentage of those genes that are in the module (% In Module), the name of the collection in MSIGDB [83] (Source), and a link to more information on the pathway (Description). There was no statistically significant pathway with *p*-value less than 10^−4^ for the modules that are not included. (XLS 131 kb)

Additional file 3
**Table S1.** Module assignments. The module assignments for all of the 9,166 genes are available as part of the online supplementary materials. The columns of the table include:  • Symbol: The gene name.  • ENTREZ ID: The gene ID in the Global Query Cross-Database Search System.  • Probe representative: The representative probe mapped to this gene.  • Adjusted p-value: For the null hypothesis that expression is similar in AML and MDS (Supplementary Note 2).  • Module ID: The assigned module.  • IMC: Intramodular connectivity.  • EMC: Extramodular connectivity.  • Rank: The rank of the gene in the module based on its intramodular connectivity.  • # of references The number of studies that reported the gene being relevant to AML according to Miller et al,. survey [81]. Intramodular and extramodular connectivities were computed by intramodularConnectivity() function from WGNCA package. They measure the overall correlation of the gene with other genes within and outside the module, respectively. The genes with the most connectivity in a module are considered as the “hubs” of that module [82]. See WGNCA manual for more information. (XLS 1618 kb)

Additional file 4
**Table S3.** Enrichment in AML-associated genes. The enrichment of modules in genes associated with AML is reported as part of the online supplementary materials. Miller et al,. systematically surveyed 25 published reports of gene expression profiling in AML [81]. We used this survey to score the modules based on their know association with AML. For instance, the # of Reported Genes-2 and Score-2 columns respectively report the number and the percentage of genes in each module that were reported to be related to AML in at least 2 studies according to Miller et al,. survey. The Module ID, Module Size, and the most overrepresented pathway in each module are also reported. These data are plotted in Additional file 1: Figure S4. (XLS 39.5 kb)

Additional file 5
**Table S4.** Eigengenes. The eigengenes computed for all gene modules are available as part of the online supplementary materials. Each row reports the values for a sample and columns correspond to modules. The first sheet was computed using moduleEigengenes function from WGCNA package, which applied PCA on the gene expression in the MILE dataset. The second sheet shows the inferred expression of these 33 eigengenes in the BCCA dataset (Methods). Module zero contains the set of “outlier” genes that did not correlated with each other or with the rest of the genome. WGCNA could not confidently assign them to any module and we did not use them in our analysis. (XLS 334 kb)

Additional file 6
**Table S5.** Classification results. The results of classification of the BCCA and the MILE cases using our decision tree are available as part of the online supplementary materials. The first sheet reports classification results of 176 BCCA patients. For each case sample ID, age, gender and the corresponding cohort are reported. The other columns of the table include:  • Sequenced Tissue: Bone marrow (BM) or peripheral blood (PB).  • RIN: The RNA Integrity Number, a value in the range of 1–10 measuring the quality of the RNA samples [84]. A higher value corresponds to less degradation of RNA.  • Subtype: The AML subtypes were diagnosed based on chromosomal abnormalities and other factors. AML-NK stands for AML with normal karyotype. tAML and tMDS are therapy related.  • Type: All AML subtypes were labeled as AML. MDS and tMDS were labeled as MDS, and 21 gray-highlighted cases that had clinical and pathological characteristics of both diseases were labeled as AML-MDS.  • Prediction (Full Tree) The label predicted by the decision tree using the inferred extracellular matrix and HOXA&B eigengenes. relevant misclassified.  • Prediction (Shrunk Tree) The label predicted by the shrunk decision tree using expression of only 14 genes from the extracellular matrix and HOXA&B modules.  • Signature The leaf of the full decision tree determined based on the expression of the extracellular matrix (ECM) and HOXA&B eigengenes. Similarly, the second sheet reports classification results of 366 patients from the MILE dataset. Additional information such as IPSS, blast, and karyotype categories are included from MILE study [16]. The misclassified cases by either the full or the shrunk tree are highlighted in yellow. (XLS 155 kb)

Additional file 7
**Table S7.** The extracellular matrix module. For each gene in the extracellular matrix module, the cellular component ontology (GO-CC) is available as part of the online supplementary materials. The genes are ordered according to their weight (also known as “membership” [85]) in the module eigengene, e.g., the first gene has the highest contribution to the eigengene. See the description of Additional file 2: Table S1 for the definition of other columns. (XLS 37 kb)

Additional file 8
**Table S9.** Biological processes. We used PANTHER (Version 10) [41] to identify the GO biological processes that are enriched in the 113 genes in our extracellular module. The columns of the resulting table include the name of GO biological process, the number of genes in the corresponding category (Homo sapiens), the number of overlapping genes, the expected overlap, the fold enrichment, and the Bonferroni-adjusted *p*-value. (XLS 20 kb)

Additional file 9 Supplementary Data 1. DNA-methylation. The DNA methylation of *MMP9*, *MMP8*, and *MMP25* genes in TCGA dataset are available as part of the online supplementary materials. The two sheets include the patient barcodes for 194 AML and 368 control samples. The *β* values were reported at cg04656101 (equivalent to chr20:44,645,014 in hg19), cg01092036 (chr11:102,595), and cg02680314 (chr16:3,097,388), respectively (Additional file 1: Figure S9b). (XLS 102 kb)

Additional file 10
**Table S8.** Validating coexpression patterns in 5,210 datasets. The descriptions and names of 5,210 datasets used to perform coexpression analysis are available as part of the online supplementary materials. SEEK sorted the datasets based on their coexpression score computed using the 113 genes from our extracellular matrix module. The AML-related datasets, highlighted in yellow, are frequent at the top of the list. The details of coexpression score and *p*-value computation are defined in the corresponding publication [44]. (XLS 1044 kb)

Additional file 11
**Table S6.** The cell cycle and translational control modules. Two modules with 319 and 193 genes were automatically selected by breast cancer survival analysis. In each sheet, gene symbols, Entrez IDs, and weights in the corresponding modules are reported. Genes are sorted based on their weights. (XLS 55 kb)

Additional file 12 Supplementary Code 1. Geo2R. The Geo2R script, used to compute *p*-values for probes, is available as part of the online supplementary materials. The process starts by downloading MILE data from GEO. (TXT 5.91 kb)

## Abbreviations

AML: Acute myeloid leukemia
AML–NK AML: With normal karyotype
BCCA: British Columbia cancer agency
GEO: Gene expression omnibus
GO–CC: Gene ontology cellular component
HSC: Hematopoietic stem cells
IPSS: Prognostic scoring system
MDS: Myelodysplastic syndrome
METABRIC: Molecular taxonomy of breast cancer international consortium
MILE: Microarray innovations in leukemia
PCA: Principal component analysis
RPKM: Reads per kilobase per million mapped reads
SEEK: Search-based exploration of expression compendium
TCGA: The cancer genome atlas
