# Supplementary material for: Large-scale gene network analysis reveals the significance of extracellular matrix pathway and homeobox genes in acute myeloid leukemia: an introduction to the Pigengene package and its applications
Source: BMC Med Genomics. 2017 Mar 16;10:16. doi: 10.1186/s12920-017-0253-6 (PMC5353782; doi:10.1186/s12920-017-0253-6)
Supplement: Additional file 1 — Supplementary Notes and Figures. (PDF 1656 kb) [file 12920_2017_253_MOESM1_ESM.pdf]

Large-scale gene network analysis reveals the significance of extracellular matrix pathway and homeobox genes in acute myeloid leukemia: an introduction to the Pigengene package and its applications

Amir Foroushani[1], Rupesh Agrahari[1], Roderick Docking[2], Linda Chang[2], Gerben Duns[2], Monika Hudoba[3], Aly Karsan[2,†*], and Habil Zare[1,†*]

[1]Department of Computer Science, Texas State University, San Marcos, TX, US.
[2]Department of Pathology and Laboratory Medicine, British Columbia Cancer Agency, Vancouver, BC, Canada.
[3]Department of Pathology and Laboratory Medicine, Vancouver General Hospital, Vancouver, BC, Canada.

List of Supplementary Figures

1 The distribution of module sizes. 4
2 Overrepresentation analysis on modules (part 1). 5
3 Overrepresentation analysis on modules (part 2). 6
4 Miller scores. 7
5 Expression of all eigengenes in the MILE dataset. 8
6 Comparing the expression of all genes in the extracellular matrix module. 10
7 The expression of genes in HOXA&B module in the BCCA dataset. 11
8 Overrepresented cellular component categories in the extracellular matrix module. . . . 12
9 Expression and DNA-methylation of three genes from MMP family. 13
10 Robustness. 14
11 Breast cancer survival analysis. 15
12 Association with translational control. 16

List of Supplementary Notes

Supplementary Note 1: The advantages of network analysis 2
Supplementary Note 2: Identifying gene modules 2
Supplementary Note 3: Fitting a Bayesian network to the eigengenes 9
Supplementary Note 4: Survival analysis on breast cancer 17

Supplementary Note 1: The advantages of network analysis. Biological processes in a cell often require intricate coordination between *multiple* genes and proteins, not just one gene or a single protein.[1] Therefore, the traditional approaches that study associations between individual genes and specific diseases are unable to provide a full understanding of complex biological phenomena.[2]

Although statistical tests such as ANOVA,[3–6] t-test,[7,8] and rank products[9–11] are successfully used in many studies to identify deferentially expressed genes,[11–15] they have a limited statistical power.[2] These approaches fail to pinpoint the biological mechanisms of complicated conditions such as cancer because complex diseases are usually caused by collaboration of more than a few genes.[16] In other words, subtle but coordinated and consistent changes in the expression of a set of functionally related genes can be more important and informative than dramatic changes in the expression of a few individual genes.[1]

Gene network analysis models the interactions between genes in a comprehensive structure.[17,18] The approaches taken to infer gene or protein networks[19–21] include ordinary differential equations (ODEs),[22–25] Boolean networks,[26,27] cofunction networks,[28–32] coexpression analysis,[33–41] and Bayesian networks.[42–48] Our methodology is inspired by, and builds upon, Bayesian network and coexpression network analyses that have been successfully used for interpreting many biological experiments. While coexpression analysis can potentially model the entire genome, unfortunately, its application is limited due to low accuracy, a deficiency that is rooted in imperfect clustering.[49,50] Bayesian networks can accurately model complicated probabilistic dependencies between a handful of genes. However, it is very challenging to fit them to the data when the number of genes exceeds hundreds or thousands.[43,51] We addressed this challenge by comparing expressions at the module level. Our analysis is robust to biological and technical noise because an eigengene is a weighted *average* expression of several genes, and thus, not affected by random fluctuation in expressions of a few genes (Supplementary Fig. 10).

Supplementary Note 2: Identifying gene modules. The MILE dataset includes expression data of 202 AML and 164 MDS cases measured using Affymetrix microarrays with 54,000 probes (see Methods). Using GEO2R web application[a],[52] we obtained an R script that ranked all of the 54,000 probes based on their p-values. The script used limma[53] (version 3.22.7) to test for each probe the null hypothesis that it was similarly expressed in AML and MDS (Supplementary Script 1). Consistent with the approach taken by other scholars,[43] we kept all of the top one-third (n=18,200) of the most variable probes in our analysis.

We used Custom CDF[54] (version 15) to map probes to Entrez-gene IDs. The mapping was not one-to-one and we took the following approach to project the data from the probe level to the gene level. First, we excluded the probes that were mapped to multiple Entrez-gene IDs. Out of 18,200 probes, 13,294 remained. Next, among all probes that were mapped to a specific Entrez-gene, the one with the lowest p-value was chosen as the “representative” of that gene. That is, we considered the most differentially expressed probe as the representative of a gene. For genes with only one corresponding probe, this probe was taken as the representative. Our approach resulted in an expression profile with 9,166 probes, each representing to a unique Entrez-gene

[a] http://www.ncbi.nlm.nih.gov/geo/geo2r/?acc=GSE15061

with expression values for 202 AML and 164 MDS cases. We stored these data in two $9,166 \times 202$ and $9,166 \times 164$ matrices.

To identify the gene modules, we applied Weighted correlation network analysis (WGCNA, version 1.41) on the $9,166 \times 202$ AML expression matrix[35] (Supplementary Table 1). Specifically, we used the following parameters to call `blockwiseModules()` function from WGCNA package. The `power` (β) parameter was adjusted based on the recommendation of authors using `pickSoftThreshold()` function with the default value of `RsquaredCut=0.85`. For better results, we set the parameter `maxBlockSize=9166` so that the process was done in only one block. We set `TOMType= "unsigned"`, and used the default values for the rest of the arguments of `blockwiseModules()`. WGCNA could not confidently assign 4,125 genes to any of the modules because they hardly correlated with any other gene. They were designated as module 0, and excluded from the rest of the analysis.

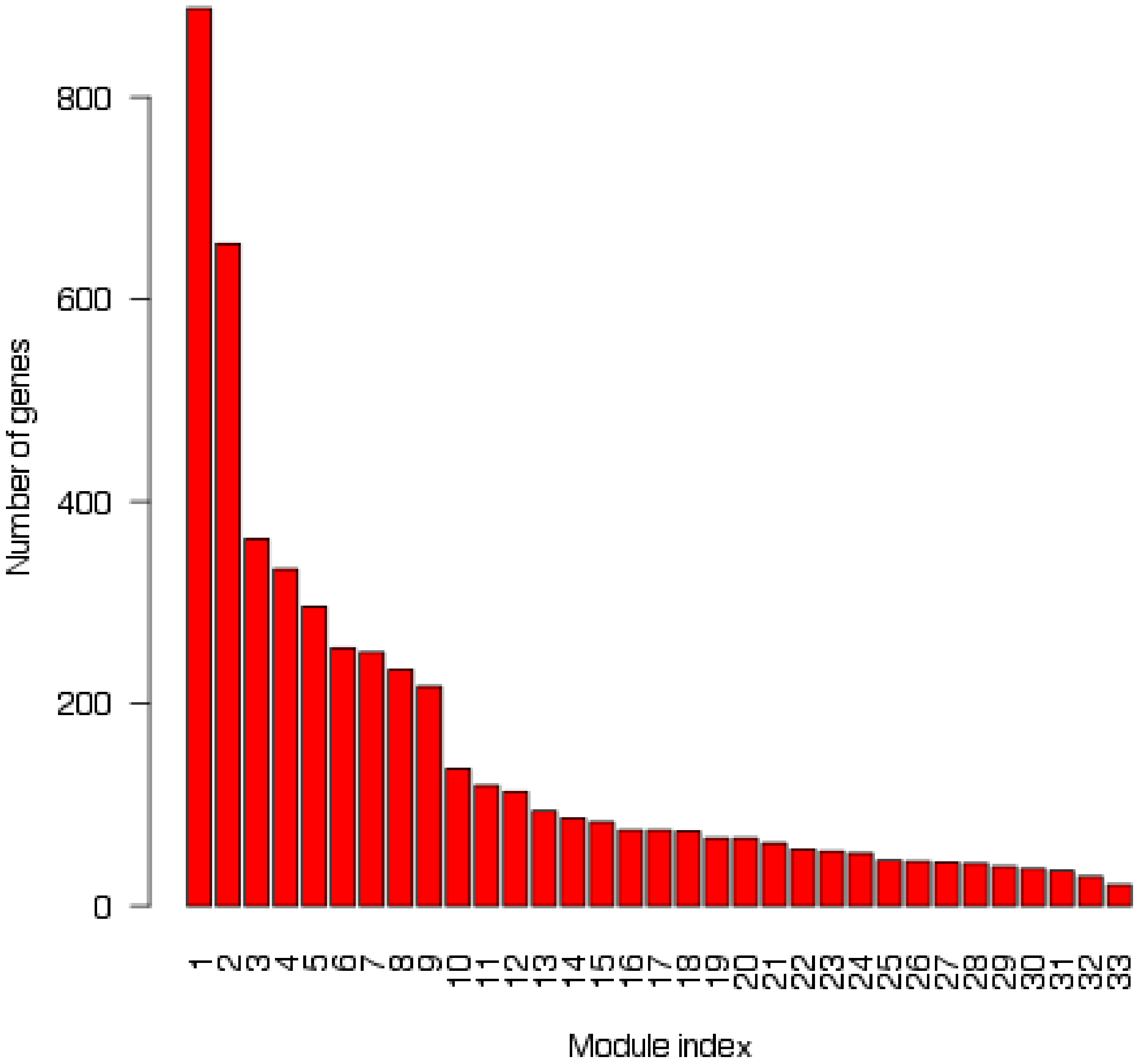

Supplementary Figure 1. The distribution of module sizes. The 33 modules are sorted on the x-axes based on their sizes. The largest and smallest modules consist of 888 and 21 genes, respectively. Module sizes had a mean, median, and standard deviation of 153, 75, and 188, respectively. The plot does not include module 0, the set of 4,125 outlier genes that WGCNA could not confidently assigned to any module.

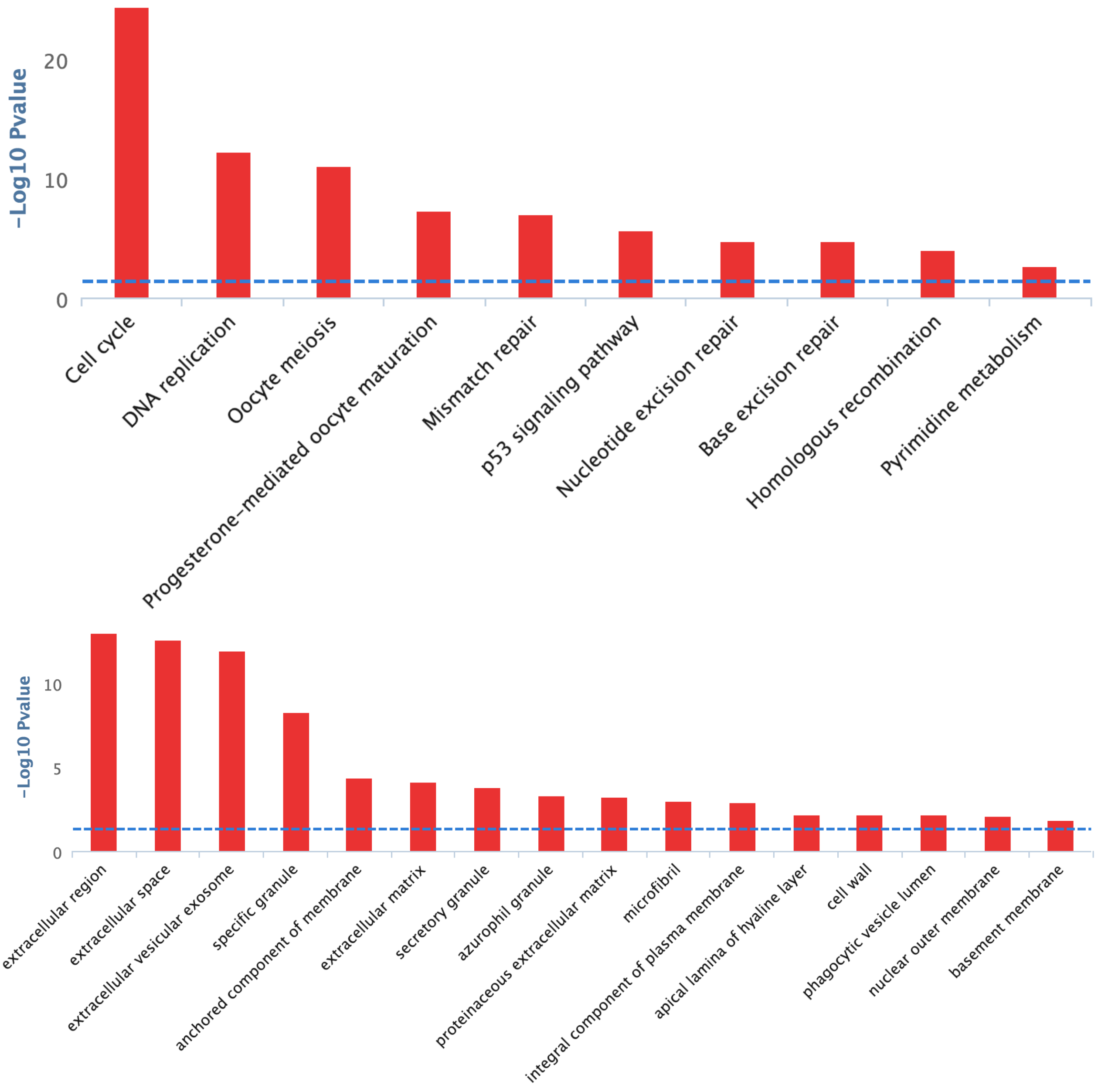

Supplementary Figure 2. Overrepresentation analysis on modules (part 1). Module 6 is associated with the cell cycle (**a**), and module 12 with the extracellular region (**b**). The dashed blue line indicates an adjusted p-value of 0.05. The plots are produced using InnateDB website.[55]

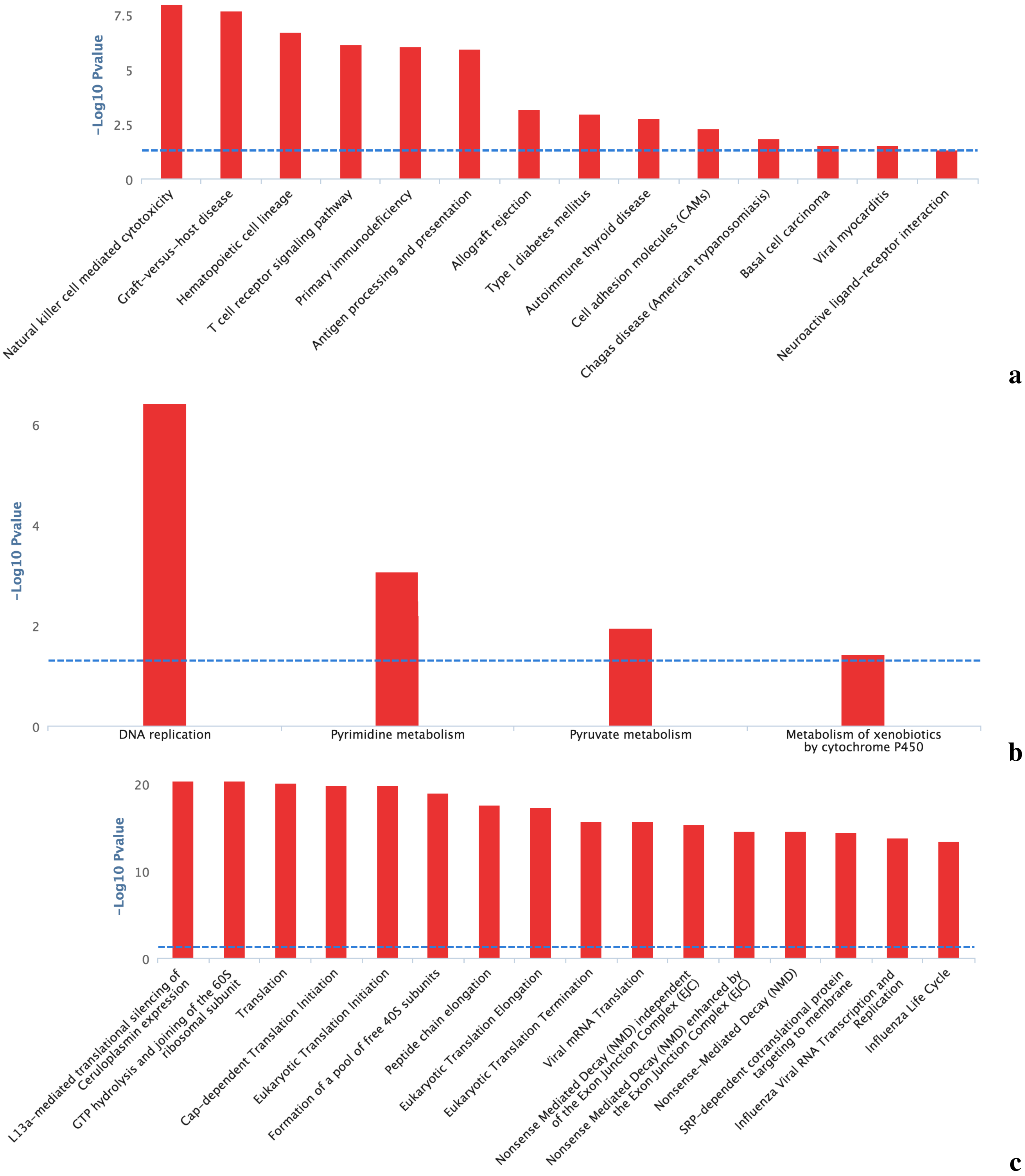

Supplementary Figure 3. Overrepresentation analysis on modules (part 2). Module 14 is associated with cytotoxic pathway **(a)**, Module 15 with DNA replication **(b)**, and module 21 with translation **(c)**.

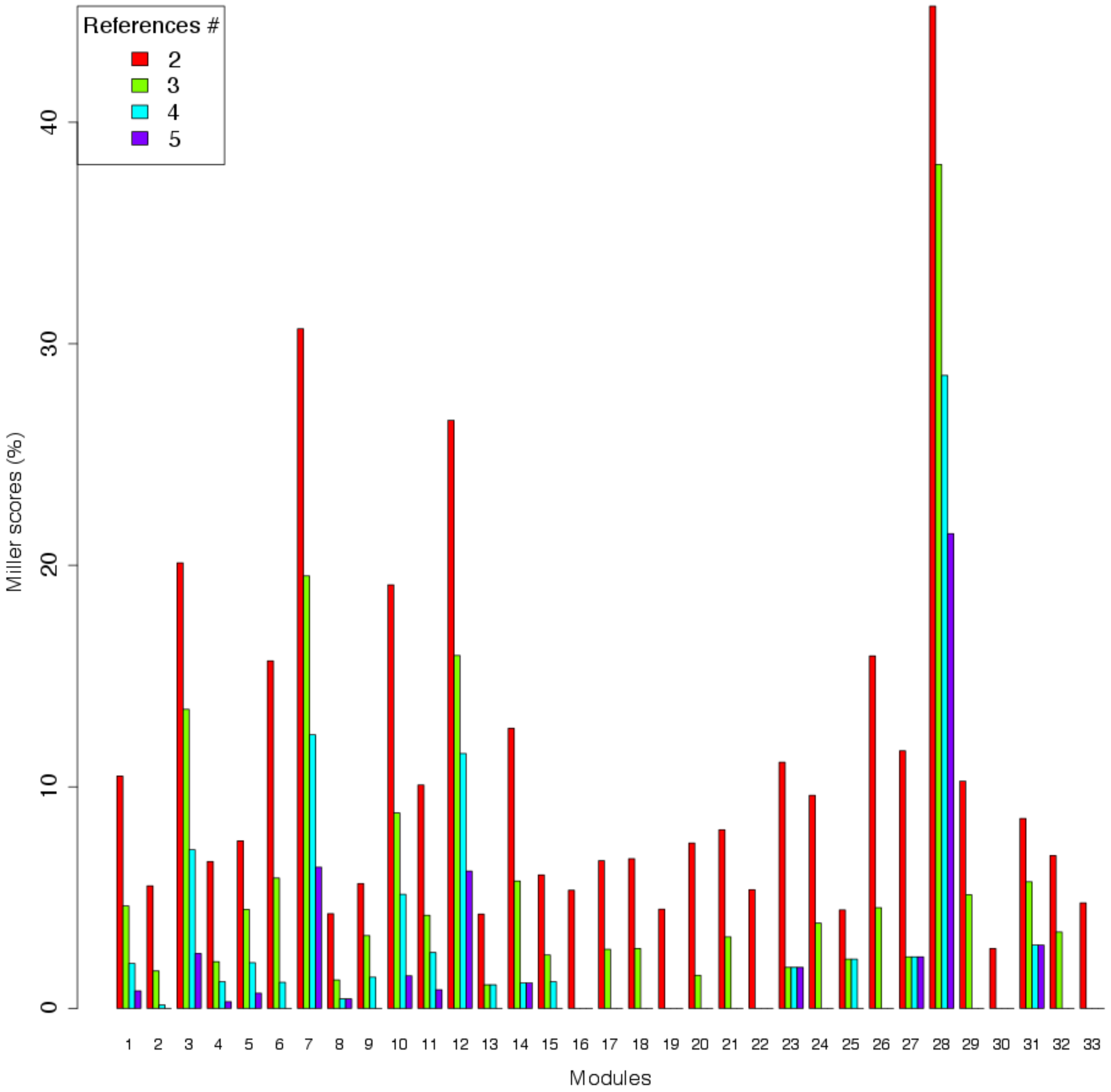

Supplementary Figure 4. Miller scores. The 33 modules identified using the MILE dataset are sorted on the x-axes based on their sizes. The y-axis shows the percentage of genes in each module that were reported to be related to AML in at least 2 (red), 3 (green), 4 (blue), and 5 (purple) studies according to Miller et al. survey.[56] The HOXA&B module (28) had the highest enrichment as expected because many of *HOXA* and *HOXB* genes were reported by scholars to be associated to AML.[56–74] Supplementary Table 3 includes the numbers corresponding to this plot.

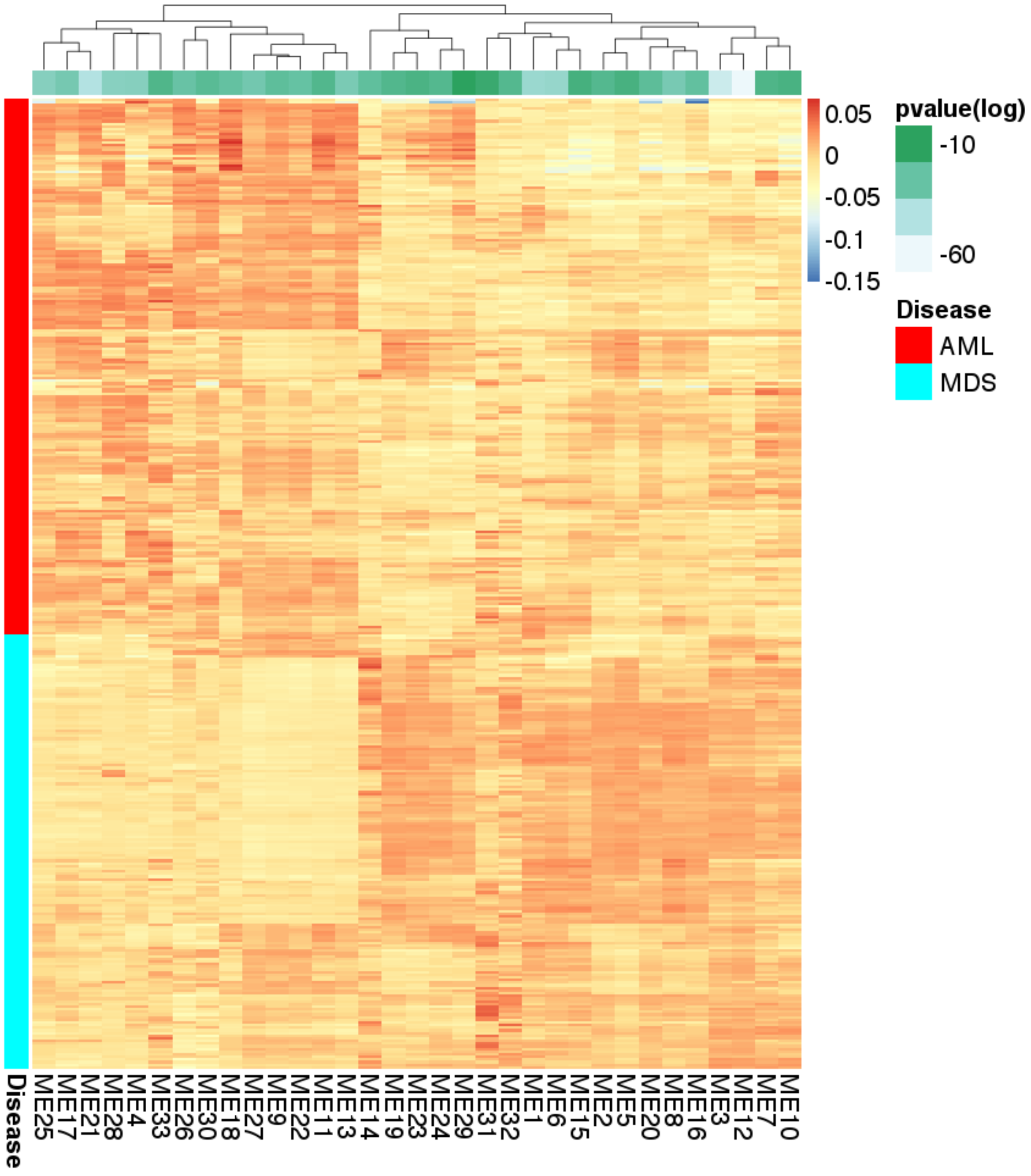

Supplementary Figure 5. Expression of all eigengenes in the MILE dataset. Eigengenes show significantly different pattern in the samples (rows) for the two disease types in the MILE data set. The green strip at the top represents the logarithm of adjusted p-values in base 10. Columns (modules) are clustered based on the similarity of expression in the MILE dataset. The expressions of these eigengenes in the MILE and BCCA datasets are reported in Supplementary Table 4.

Supplementary Note 3: Fitting a Bayesian network to the eigengenes.

A Bayesian network is a statistical model that represents a set of random variables using a directed acyclic graph.[75] Nodes of the network correspond to random variables and the edges (arcs) model their conditional dependencies. An important property of Bayesian networks is that each node conditioned on its parent variables is independent of its non-descendants. In particular, if two nodes are not connected by a directed path, they are conditionally independent.

We trained a Bayesian network to model the probabilistic dependencies between the modules. Each module eigengene was represented by a node (observed random variable). To model the hematological malignancy, we added "Disease" as an observed random variable to the network. For instance in our study, it was equal to 1 for AML, and 0 for MDS. No eigengene was allowed to be a parent of Disease node.

We used bnlearn package to infer the edges and fit the above Bayesian network to the eigengenes.[76] Specifically, we discretized the values of eigengenes into three levels using Hartemink's method.[77] We used the `bn.boot()` function from the bnlean package to fit 1000 networks to the discretized data. This function used hill climbing strategy to optimize Bayesian Dirichlet equivalent (BDe) score.[78] Consistent with the approach taken by other scholars,[43] we averaged one-third of the notworks with the highest scores to obtain the consensus network. To facilitate applying the above procedure in other studies, we provided `learn.bn()` function in our Pigengene R package (version 0.99.19). In particular, Code 1 reproduces the results presented in this paper. See the package manual for more detail.

Code 1. Reproducing the Bayesian network

```
library(Pigengene) ## version 0.99.19
data(eigengenes33)
amlE <- eigengenes33$aml
mdsE <- eigengenes33$mds
eigengenes <- rbind(amlE,mdsE)
Labels <- c(rep("AML",nrow(amlE)),rep("MDS",nrow(mdsE)))
names(Labels) <- rownames(eigengenes)
learnt <- learn.bn(Data=eigengenes, Labels=Labels,
  bnPath="bn", bnNum=1000, seed=1, verbose =4)
## Visualize:
d1 <- draw.bn(BN=learnt$consensus1$BN,nodeFontSize=18)
```

The computation does not need more than 2 MB of memory and it is done in one to two days depending on the computer speed. Our package is capable of learning the networks in parallel using a computer cluster. Parallelization results in decreasing the wall-time substantially, dividing it by the number of available compute nodes.

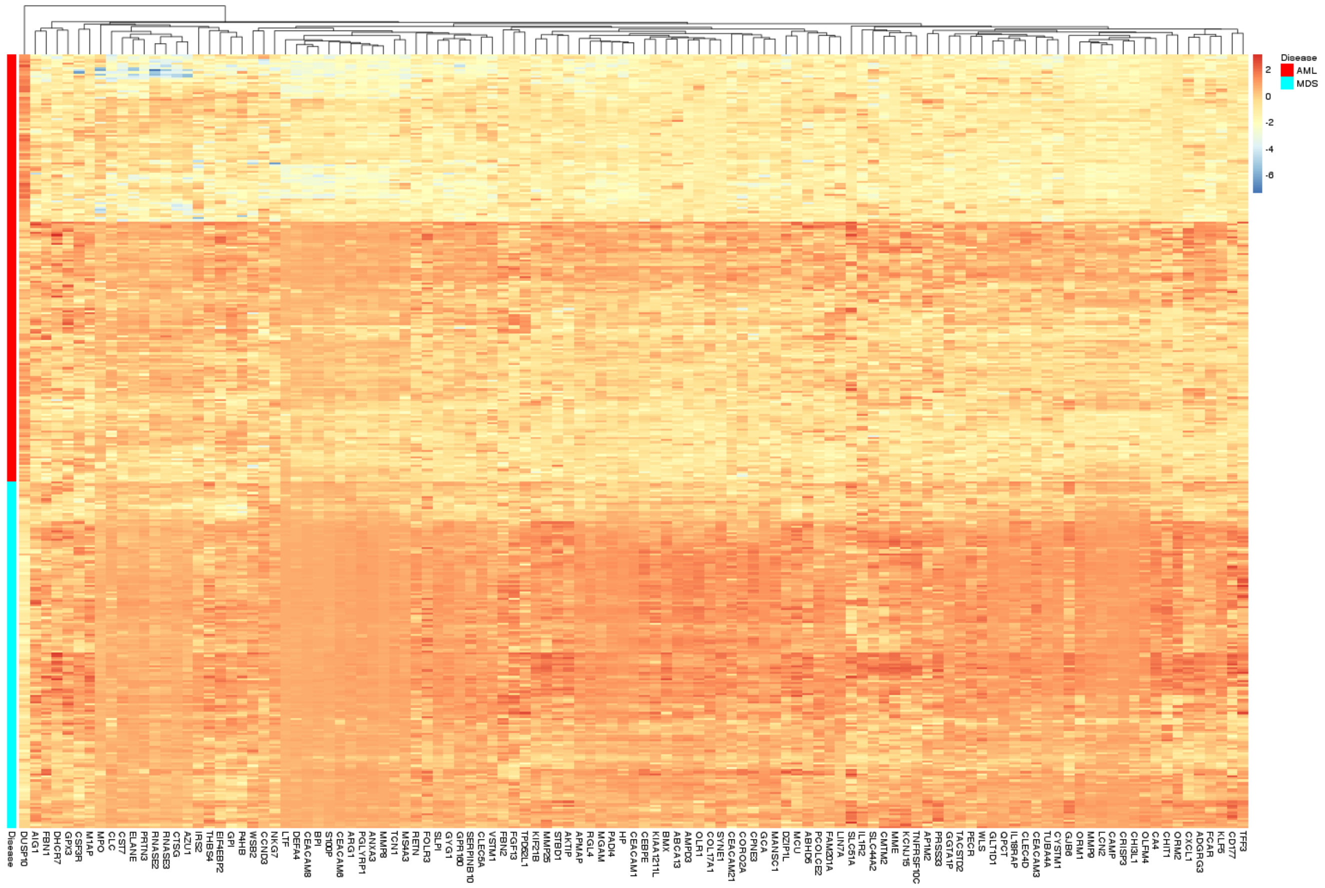

Supplementary Figure 6. Comparing the expression of all genes in the extracellular matrix module. Expressions of every member of module 12, the module associated with the extracellular matrix, are shown in a column. Similar to the extracellular region subset (Fig. 5), these genes are mostly underexpressed in AML compared to MDS. Their variable expressions in some AML cases indicate the heterogeneity of the disease. They have positive weights in the corresponding eigengene except DUSP10 (weight= −0.8).

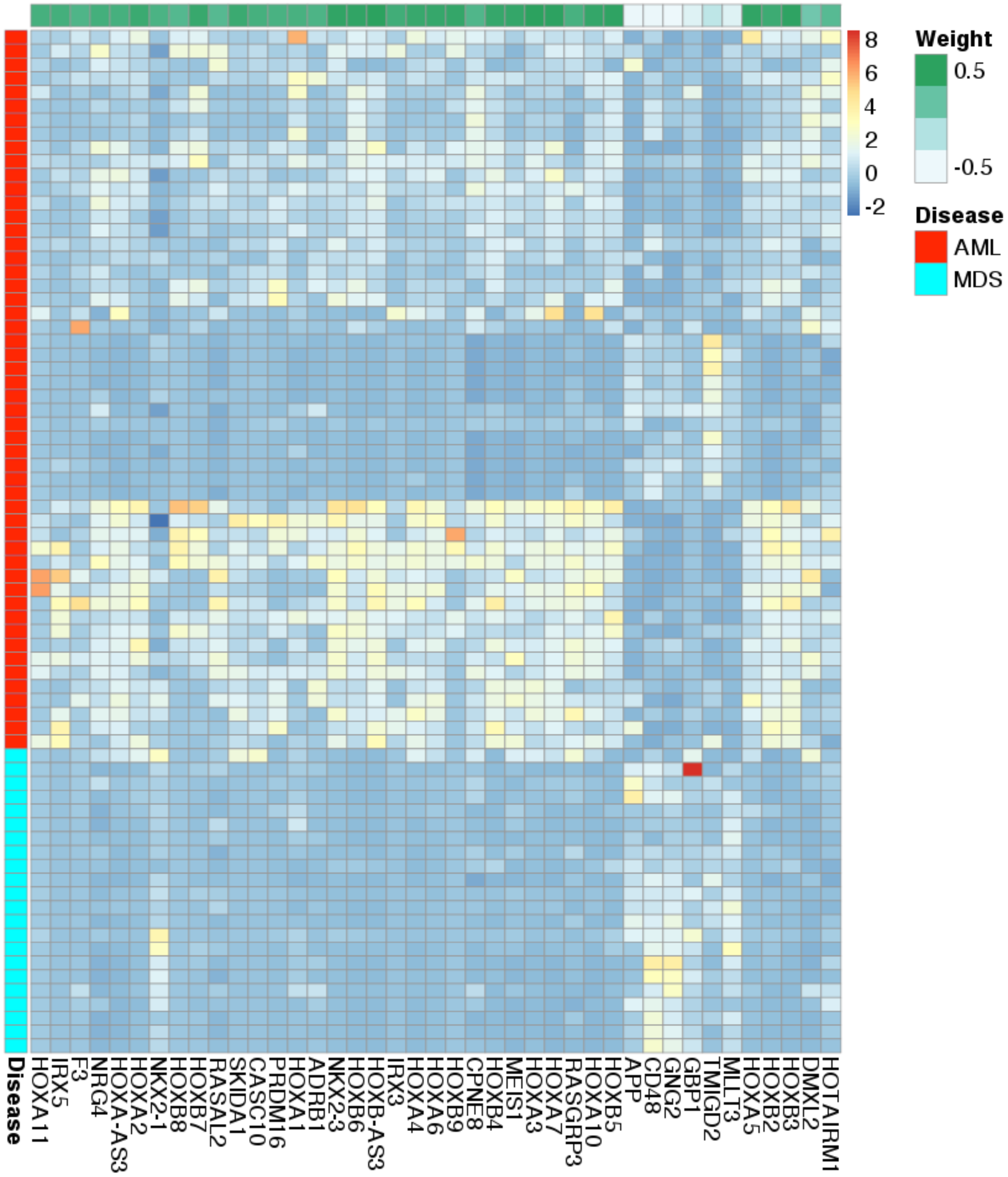

Supplementary Figure 7. The expression of genes in HOXA&B module in the BCCA dataset. The pattern of gene expressions is similar to the MILE dataset, i.e., the majority of these HOXA and HOXB genes are not expressed in MDS. For clarity, the columns are scaled, and have the same ordering as in Fig. 6.

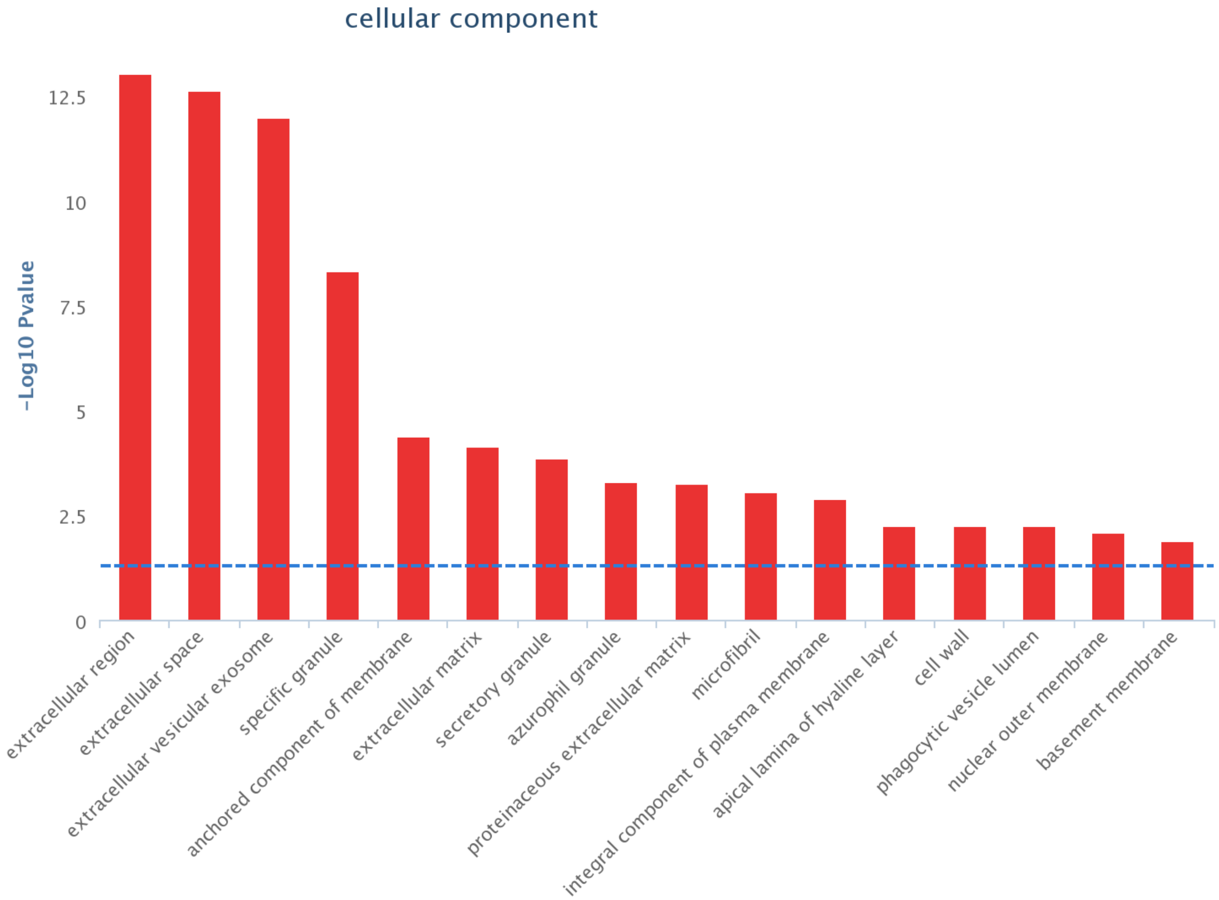

Supplementary Figure 8. Overrepresented cellular component categories in the extracellular matrix module. The extracellular region is the most overrepresented category in module 12, followed by the extracellular space and the extracellular vesicular exosome. From the 113 members of this module, 36 (32%) genes code for proteins in the extracellular region, which is a category with 1,525 genes, 8% of total number of human genes (adjusted p-value $\leq 7 \times 10^{-11}$).

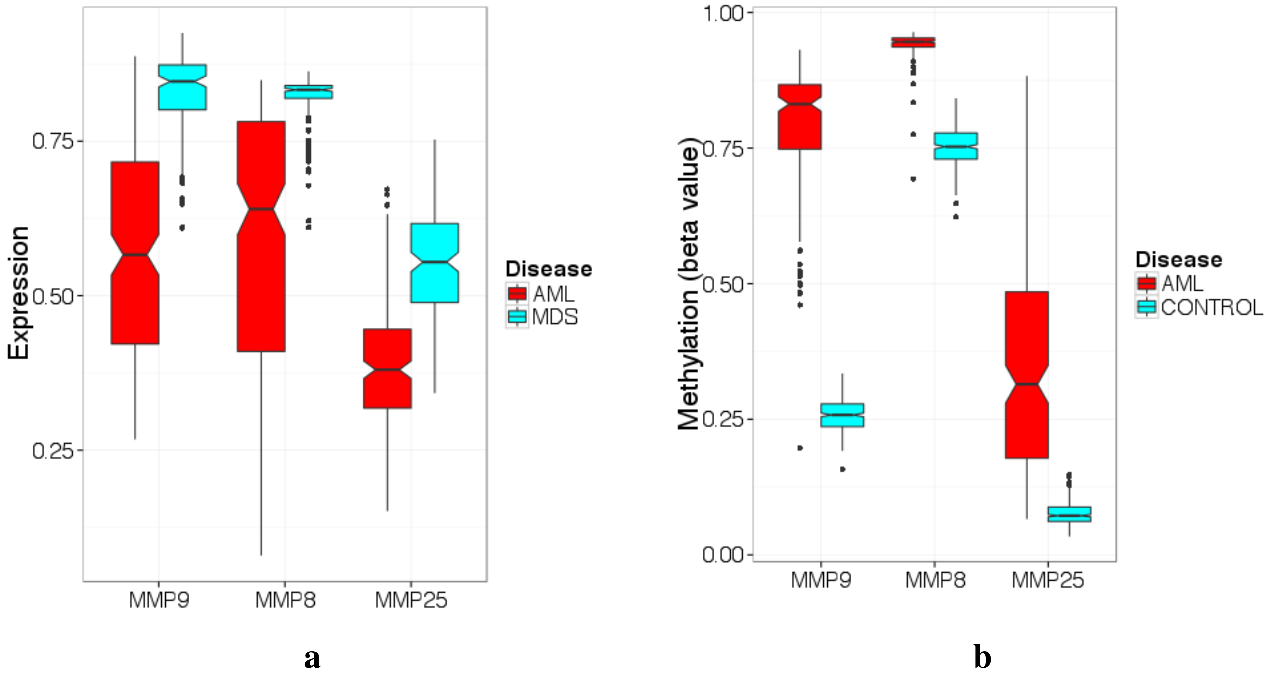

Supplementary Figure 9. Expression and DNA-methylation of three genes from MMP family. *MMP9*, *MMp8*, and *MMP25* are the three genes from the matrix metalloproteinase (MMP) family that have relatively high contribution to our extracellular matrix eigengene. **(a)** They are significantly underexpressed in AML compared to MDS (adjusted p-values in the MILE dataset $\leq 10^{-53}$, 10^{-32}, and 10^{-38}, respectively.) **(b)** The y-axis shows the β value, which corresponds to the percentage of DNA-methylation in each sample.[79] For each gene, methylation at a locus close to its promoter is shown (Supplementary Data 1). These three genes are heavily methylated in the majority of 194 AML cases (red) in comparison to 368 control cases (blue), Welch's t-test p-values are 10^{-138}, 10^{-252}, and 10^{-46}, respectively.

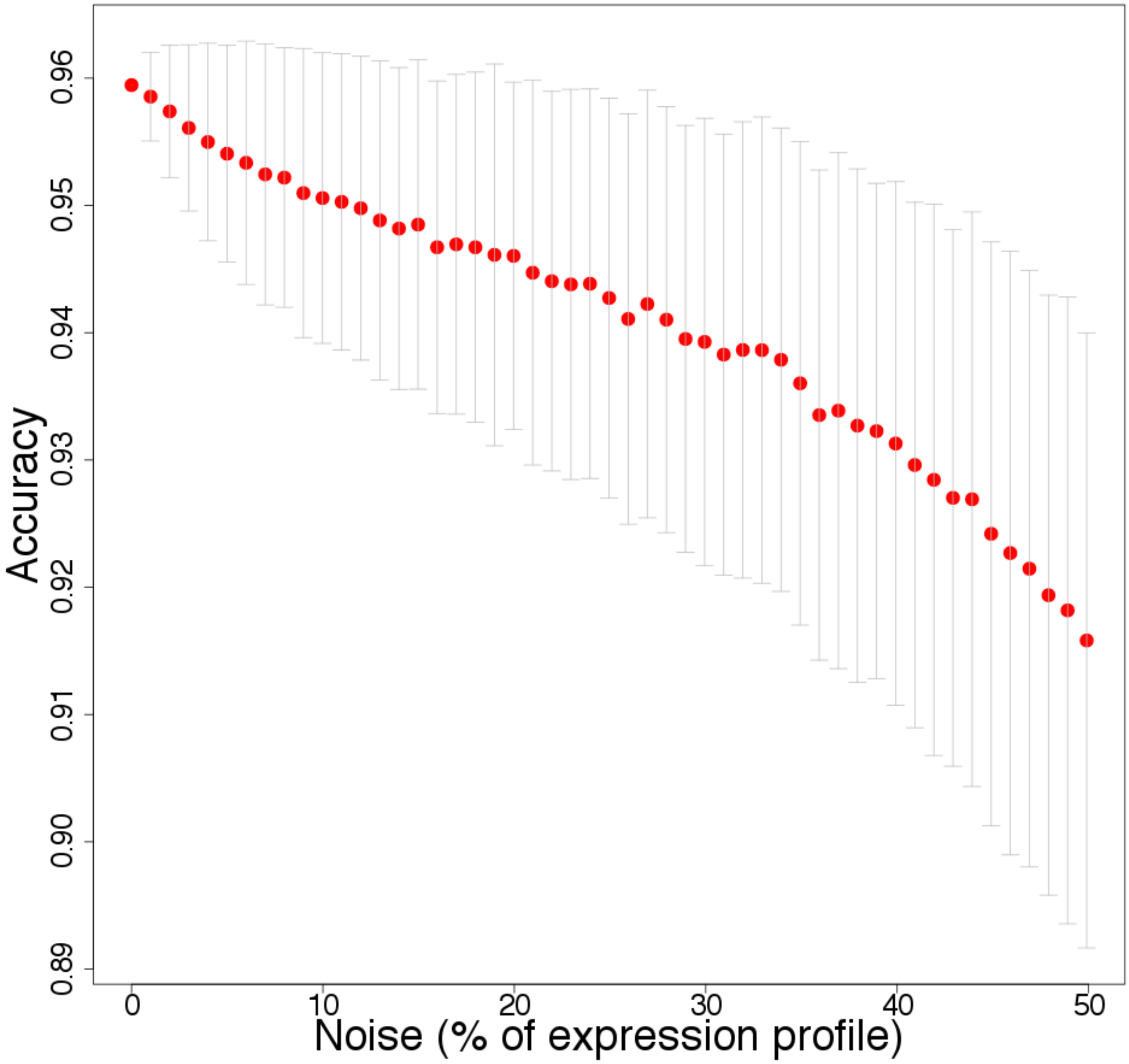

Supplementary Figure 10. Robustness. To quantify the affect of noise on our analysis, we replaced random entries of the expression profile from the BCCA dataset with Gaussian noise, $\mathcal{N}(\mu,\rho)$, where $\mu = 0.008$ and $\rho = 0.95$ are the mean and the standard deviation of expression data, respectively. The x-axis shows the percentage of perturbed entries in the expression profile. The average accuracy of our decision tree over 1000 runs are shown on the y-axis. The error bars correspond to the standard deviation. The accuracy is very robust with respect to noise, for instance, even when 30% of the expression profile is perturbed, the decline in the accuracy is negligible (2%).

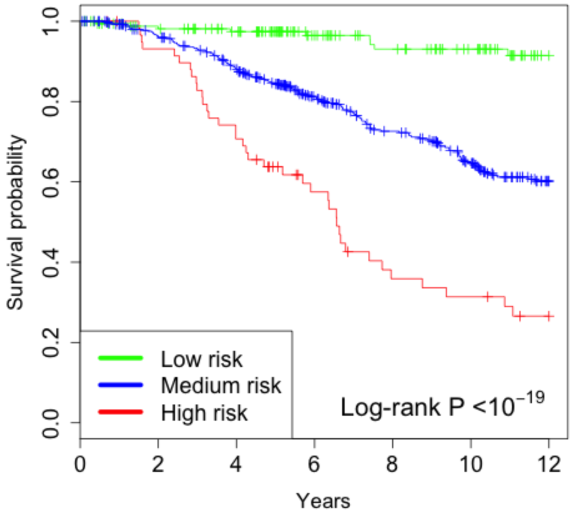

a

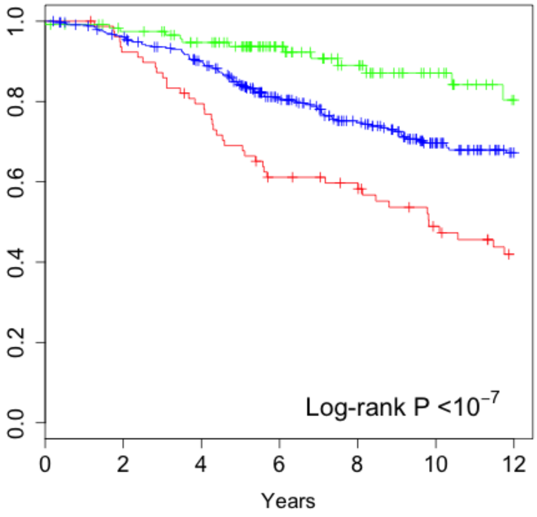

b

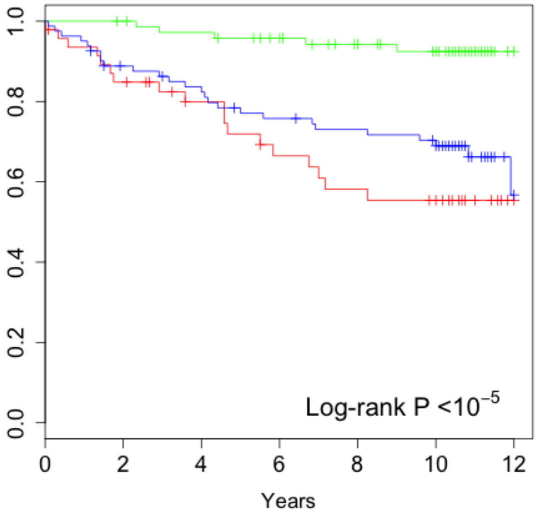

c

Supplementary Figure 11. Breast cancer survival analysis. Kaplan–Meier survival curves are shown for the three groups of ER+ patients classified by our survival analysis (Supplementary Note 4). Each plot corresponds to a dataset. We identified the biological signatures using METABRIC training dataset (**a**), and confirmed their predictive value in METABRIC validation (**b**) and MILLER (**c**) datasets (Methods). The low-risk patients are identified by regulated cell cycle and transcription (green). For this group, the probability of surviving more than 10 years is above 89% in all the three datasets. The p-values indicate that the difference between low and high risk groups is statistically significant.

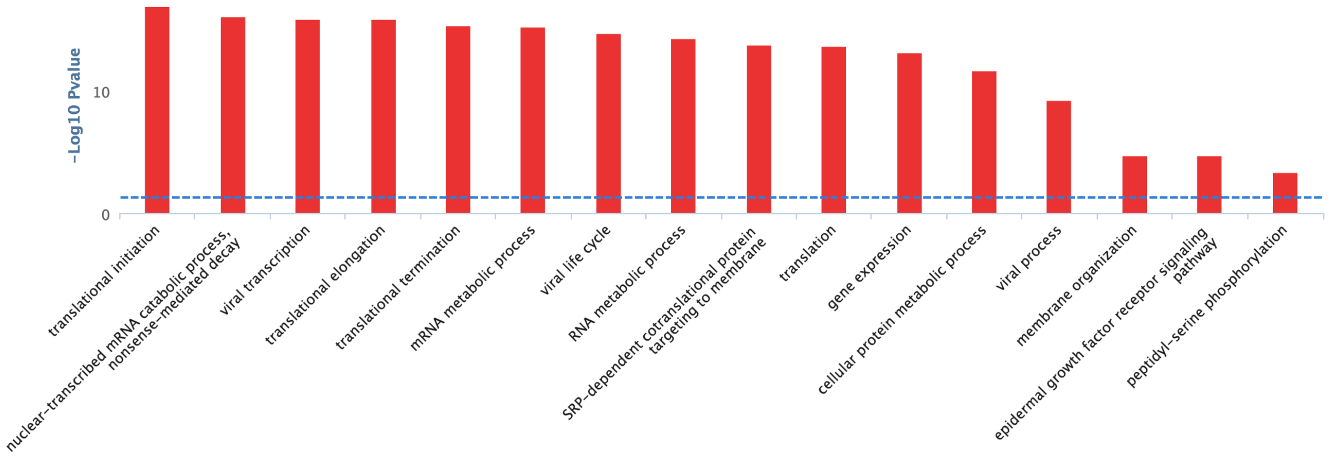

Supplementary Figure 12. Association with translational control. The y-axis shows the p-value from overrepresentation analysis on the smaller module that was automatically-selected by breast cancer survival analysis. This module of 193 genes is significantly associated with translation and translational control.

Supplementary Note 4: Survival analysis on breast cancer. We applied a methodology similar to our AML-MDS analysis on METABRIC discovery dataset to identify 15 modules and compute the corresponding eigengenes. We used glmnet package (version 2.0-2) to fit a regularized Cox model with the Lasso penalty ($\alpha = 1$).[80] The regularization path indicated that two modules with 319 and 193 genes are most associated with survival. We used the corresponding two eigengenes to fit an accelerated failure time model to the survival data. Specifically, we used `survreg` function from Survival package (version 2.38-3), Weibull distribution with `scale=1`, and the defaults values for the rest of the parameters.[81] We used the fitted model to predict the survival time using only the two eigengenes. We chose two thresholds for the predicted values that maximized the precision of low and high risk predictions in the METABRIC discovery dataset. We inferred the two eigengenes in METABRIC validation and MILLER datasets to evaluate our accelerated failure time model. Using the same thresholds, our approach could identify low-risk patients in these two independent datasets with high specificity ($> 89\%$, Table 3 and Supplementary Fig. 11). This illustrates the biological significance of the identified signatures.

References

1. Cho, D.-Y., Kim, Y.-A. & Przytycka, T. M. Network biology approach to complex diseases. *PLoS Comput Biol* **8**, e1002820 (2012).
2. Halsey, L. G., Curran-Everett, D., Vowler, S. L. & Drummond, G. B. The fickle p value generates irreproducible results. *Nature methods* **12**, 179–185 (2015).
3. Cui, X., Churchill, G. A. *et al.* Statistical tests for differential expression in cdna microarray experiments. *Genome Biol* **4**, 210 (2003).
4. Wolfinger, R. D. *et al.* Assessing gene significance from cdna microarray expression data via mixed models. *Journal of Computational Biology* **8**, 625–637 (2001).
5. Kerr, M. K. & Churchill, G. A. Analysis of variance for gene expression microarray data. *Journal of Computational Biology* **7**, 819–837 (2000).
6. Tan, P. K. *et al.* Evaluation of gene expression measurements from commercial microarray platforms. *Nucleic acids research* **31**, 5676–5684 (2003).
7. Huber, W., Von Heydebreck, A., Sültmann, H., Poustka, A. & Vingron, M. Variance stabilization applied to microarray data calibration and to the quantification of differential expression. *Bioinformatics* **18**, S96–S104 (2002).
8. Baggerly, K. A. *et al.* Identifying differentially expressed genes in cdna microarray experiments. *Journal of Computational Biology* **8**, 639–659 (2001).
9. Breitling, R., Armengaud, P., Amtmann, A. & Herzyk, P. Rank products: a simple, yet powerful, new method to detect differentially regulated genes in replicated microarray experiments. *FEBS letters* **573**, 83–92 (2004).
10. Jeffery, I. B., Higgins, D. G. & Culhane, A. C. Comparison and evaluation of methods for generating differentially expressed gene lists from microarray data. *BMC bioinformatics* **7**, 359 (2006).
11. Subramanian, A. *et al.* Gene set enrichment analysis: A knowledge-based approach for interpreting genome-wide expression profiles. *Proceedings of the National Academy of Sciences of the United States of America* **102**, 15545–15550 (2005).
12. Soneson, C. & Delorenzi, M. A comparison of methods for differential expression analysis of rna-seq data. *BMC bioinformatics* **14**, 91 (2013).
13. Oshlack, A., Robinson, M. D., Young, M. D. *et al.* From rna-seq reads to differential expression results. *Genome biol* **11**, 220 (2010).
14. Fortunel, N. O. *et al.* Comment on " stemness: transcriptional profiling of embryonic and adult stem cells" and "a stem cell molecular signature" (i). *Science* **302**, 393–393 (2003).
15. Mootha, V. K. *et al.* Pgc-1α-responsive genes involved in oxidative phosphorylation are coordinately downregulated in human diabetes. *Nature genetics* **34**, 267–273 (2003).
16. Choi, Y. & Kendziorski, C. Statistical methods for gene set coexpression analysis. *Bioinformatics* **25**, 2780–2786 (2009).

17. Sinoquet, C. & Mourad, R. *Probabilistic Graphical Models for Genetics, Genomics and Postgenomics* (Oxford University Press, 2014).

18. Bing, H. & Xue-wen, C. bneat: a bayesian network method for detecting epistatic interactions in genome-wide association studies. *BMC genomics* **12**, S9 (2011).

19. de la Fuente, A. *Gene Network Inference*, vol. 106 (Springer, 2013).

20. De Smet, R. & Marchal, K. Advantages and limitations of current network inference methods. *Nature Reviews Microbiology* **8**, 717–729 (2010).

21. Vanunu, O., Magger, O., Ruppin, E., Shlomi, T. & Sharan, R. *Associating genes and protein complexes with disease via network propagation*. Ph.D. thesis, Publisher not identified (2009).

22. De Jong, H. & Page, M. Search for steady states of piecewise-linear differential equation models of genetic regulatory networks. *Computational Biology and Bioinformatics, IEEE/ACM Transactions on* **5**, 208–222 (2008).

23. Wang, Y., Joshi, T., Zhang, X.-S., Xu, D. & Chen, L. Inferring gene regulatory networks from multiple microarray datasets. *Bioinformatics* **22**, 2413–2420 (2006).

24. Xiong, M., Li, J. & Fang, X. Identification of genetic networks. *Genetics* **166**, 1037–1052 (2004).

25. Molinelli, E. J. *et al.* Perturbation biology: inferring signaling networks in cellular systems. *PLoS computational biology* **9**, e1003290 (2013).

26. Huang, S. Gene expression profiling, genetic networks, and cellular states: an integrating concept for tumorigenesis and drug discovery. *Journal of Molecular Medicine* **77**, 469–480 (1999).

27. Choi, M., Shi, J., Jung, S. H., Chen, X. & Cho, K.-H. Attractor landscape analysis reveals feedback loops in the p53 network that control the cellular response to dna damage. *Science signaling* **5**, ra83–ra83 (2012).

28. Taşan, M. *et al.* Selecting causal genes from genome-wide association studies via functionally coherent subnetworks. *Nature methods* **12**, 154–159 (2015).

29. Rolland, T. *et al.* A proteome-scale map of the human interactome network. *Cell* **159**, 1212–1226 (2014).

30. Lee, I., Blom, U. M., Wang, P. I., Shim, J. E. & Marcotte, E. M. Prioritizing candidate disease genes by network-based boosting of genome-wide association data. *Genome research* **21**, 1109–1121 (2011).

31. Wang, P. I. & Marcotte, E. M. It's the machine that matters: predicting gene function and phenotype from protein networks. *Journal of proteomics* **73**, 2277–2289 (2010).

32. Franke, L. *et al.* Reconstruction of a functional human gene network, with an application for prioritizing positional candidate genes. *The American Journal of Human Genetics* **78**, 1011–1025 (2006).

33. Dhaeseleer, P., Liang, S. & Somogyi, R. Genetic network inference: from coexpression clustering to reverse engineering. *Bioinformatics* **16**, 707–726 (2000).

34. Hong, S., Chen, X., Jin, L. & Xiong, M. Canonical correlation analysis for rna-seq coexpression networks. *Nucleic acids research* **41**, e95–e95 (2013).

35. Langfelder, P. & Horvath, S. Wgcna: an r package for weighted correlation network analysis. *BMC bioinformatics* **9**, 559 (2008).

36. Song, L., Langfelder, P. & Horvath, S. Comparison of coexpression measures: mutual information, correlation, and model based indices. *BMC bioinformatics* **13**, 328 (2012).

37. Choi, J. K., Yu, U., Yoo, O. J. & Kim, S. Differential coexpression analysis using microarray data and its application to human cancer. *Bioinformatics* **21**, 4348–4355 (2005).

38. Dawson, J. A., Ye, S. & Kendziorski, C. R/ebcoexpress: an empirical bayesian framework for discovering differential coexpression. *Bioinformatics* **28**, 1939–1940 (2012).

39. Wang, K. *et al.* Met=a-analysis of inter-species liver coexpression networks elucidates traits associated with common human diseases. *PLoS Comput Biol* **5**, e1000616 (2009).

40. Das, S. K., Sharma, N. K. & Zhang, B. Integrative network analysis reveals different pathophysiological mechanisms of insulin resistance among caucasians and african americans. *BMC medical genomics* **8**, 4 (2015).

41. Bunyavanich, S. *et al.* Integrated genome-wide association, coexpression network, and expression single nucleotide polymorphism analysis identifies novel pathway in allergic rhinitis. *BMC medical genomics* **7**, 48 (2014).

42. Chai, L. E. *et al.* A review on the computational approaches for gene regulatory network construction. *Computers in biology and medicine* **48**, 55–65 (2014).

43. Zhang, B. *et al.* Integrated systems approach identifies genetic nodes and networks in late-onset alzheimer's disease. *Cell* **153**, 707–720 (2013).

44. Friedman, N., Linial, M., Nachman, I. & Pe'er, D. Using Bayesian networks to analyze expression data. *J. Comput. Biol.* **7**, 601–620 (2000).

45. Smith, V. A., Yu, J., Smulders, T. V., Hartemink, A. J. & Jarvis, E. D. Computational inference of neural information flow networks. *PLoS computational biology* **2**, e161 (2006).

46. Lin, L. & Zhu, J. Using simulated data to evaluate bayesian network approach for integrating diverse data. In *Gene Network Inference*, 119–130 (Springer, 2013).

47. Isci, S., Dogan, H., Ozturk, C. & Otu, H. H. Bayesian network prior: network analysis of biological data using external knowledge. *Bioinformatics* **30**, 860–867 (2014).

48. Zacher, B. *et al.* Joint bayesian inference of condition-specific mirna and transcription factor activities from combined gene and microrna expression data. *Bioinformatics* **28**, 1714–1720 (2012).

49. Pindah, W., Nordin, S., Seman, A., Said, M. & Saifulaman, M. Review of dimensionality reduction techniques using clustering algorithm in reconstruction of gene regulatory networks.

In *Computer, Communications, and Control Technology (I4CT), 2015 International Conference on*, 172–176 (IEEE, 2015).

50. Gillis, J. & Pavlidis, P. ”guilt by association” is the exception rather than the rule in gene networks. *PLoS computational biology* **8**, e1002444 (2012).

51. Friedman, N. & Koller, D. Being bayesian about network structure. a bayesian approach to structure discovery in bayesian networks. *Machine learning* **50**, 95–125 (2003).

52. Davis, S. & Meltzer, P. S. Geoquery: a bridge between the gene expression omnibus (geo) and bioconductor. *Bioinformatics* **23**, 1846–1847 (2007).

53. Smyth, G. K. Limma: linear models for microarray data. In *Bioinformatics and computational biology solutions using R and Bioconductor*, 397–420 (Springer, 2005).

54. Dai, M. *et al.* Evolving gene/transcript definitions significantly alter the interpretation of genechip data. *Nucleic acids research* **33**, e175–e175 (2005).

55. Breuer, K. *et al.* Innatedb: systems biology of innate immunity and beyond–recent updates and continuing curation. *Nucleic acids research* gks1147 (2012).

56. Miller, B. G. & Stamatoyannopoulos, J. A. Integrative meta-analysis of differential gene expression in acute myeloid leukemia. *PLoS One* **5** (2010).

57. Alharbi, R. A., Pettengell, R., Pandha, H. S. & Morgan, R. The role of hox genes in normal hematopoiesis and acute leukemia. *Leukemia* **27**, 1000–1008 (2013).

58. Bullinger, L. *et al.* Use of gene-expression profiling to identify prognostic subclasses in adult acute myeloid leukemia. *New England Journal of Medicine* **350**, 1605–1616 (2004).

59. Alcalay, M. *et al.* Acute myeloid leukemia bearing cytoplasmic nucleophosmin (npmc+ aml) shows a distinct gene expression profile characterized by up-regulation of genes involved in stem-cell maintenance. *Blood* **106**, 899–902 (2005).

60. Mullighan, C. *et al.* Pediatric acute myeloid leukemia with npm1 mutations is characterized by a gene expression profile with dysregulated hox gene expression distinct from mll-rearranged leukemias. *Leukemia* **21**, 2000–2009 (2007).

61. Valk, P. J. *et al.* Prognostically useful gene-expression profiles in acute myeloid leukemia. *New England Journal of Medicine* **350**, 1617–1628 (2004).

62. Verhaak, R. G. *et al.* Mutations in nucleophosmin (npm1) in acute myeloid leukemia (aml): association with other gene abnormalities and previously established gene expression signatures and their favorable prognostic significance. *Blood* **106**, 3747–3754 (2005).

63. Wilson, C. S. *et al.* Gene expression profiling of adult acute myeloid leukemia identifies novel biologic clusters for risk classification and outcome prediction. *Blood* **108**, 685–696 (2006).

64. Debernardi, S. *et al.* Genome-wide analysis of acute myeloid leukemia with normal karyotype reveals a unique pattern of homeobox gene expression distinct from those with translocation-mediated fusion events. *Genes, Chromosomes and Cancer* **37**, 149–158 (2003).

65. Bullinger, L. *et al.* Gene-expression profiling identifies distinct subclasses of core binding factor acute myeloid leukemia. *Blood* **110**, 1291–1300 (2007).

66. Schoch, C. *et al.* Acute myeloid leukemias with reciprocal rearrangements can be distinguished by specific gene expression profiles. *Proceedings of the National Academy of Sciences* **99**, 10008–10013 (2002).

67. Heuser, M. *et al.* Gene-expression profiles and their association with drug resistance in adult acute myeloid leukemia. *haematologica* **90**, 1484–1492 (2005).

68. Lacayo, N. J. *et al.* Gene expression profiles at diagnosis in de novo childhood aml patients identify flt3 mutations with good clinical outcomes. *Blood* **104**, 2646–2654 (2004).

69. Radmacher, M. D. *et al.* Independent confirmation of a prognostic gene-expression signature in adult acute myeloid leukemia with a normal karyotype: a cancer and leukemia group b study. *Blood* **108**, 1677–1683 (2006).

70. Neben, K. *et al.* Distinct gene expression patterns associated with flt3-and nras-activating mutations in acute myeloid leukemia with normal karyotype. *Oncogene* **24**, 1580–1588 (2005).

71. Neben, K. *et al.* Gene expression patterns in acute myeloid leukemia correlate with centrosome aberrations and numerical chromosome changes. *Oncogene* **23**, 2379–2384 (2004).

72. Gutierrez, N. *et al.* Gene expression profile reveals deregulation of genes with relevant functions in the different subclasses of acute myeloid leukemia. *Leukemia* **19**, 402–409 (2005).

73. Ross, M. E. *et al.* Gene expression profiling of pediatric acute myelogenous leukemia. *Blood* **104**, 3679–3687 (2004).

74. Garzon, R. *et al.* Expression and prognostic impact of lncrnas in acute myeloid leukemia. *Proceedings of the National Academy of Sciences* **111**, 18679–18684 (2014).

75. Jensen, F. V. *An introduction to Bayesian networks*, vol. 210 (UCL press London, 1996).

76. Scutari, M. Learning bayesian networks with the bnlearn r package. *Journal of Statistical Software* **35**, 1–22 (2010). URL http://www.jstatsoft.org/index.php/jss/article/view/v035i03.

77. Hartemink, A. & Gifford, D. *Principled computational methods for the validation and discovery of genetic regulatory networks. Massachusetts Institute of Technology*. Ph.D. thesis, Ph. D. dissertation (2001).

78. Heckerman, D., Geiger, D. & Chickering, D. M. Learning bayesian networks: The combination of knowledge and statistical data. *Machine learning* **20**, 197–243 (1995).

79. Du, P. *et al.* Comparison of beta-value and m-value methods for quantifying methylation levels by microarray analysis. *BMC bioinformatics* **11**, 587 (2010).

80. Simon, N., Friedman, J., Hastie, T., Tibshirani, R. *et al.* Regularization paths for cox's proportional hazards model via coordinate descent. *Journal of statistical software* **39**, 1–13 (2011).

81. Kalbfleisch, J. D. & Prentice, R. L. *The statistical analysis of failure time data*, vol. 360 (John Wiley & Sons, 2011).
